# Toxic-Selenium and Low-Selenium Transcriptomes in *Caenorhabditis elegans*: Toxic Selenium Up-Regulates Oxidoreductase and Down-Regulates Cuticle-Associated Genes

**DOI:** 10.1371/journal.pone.0101408

**Published:** 2014-06-27

**Authors:** Christopher J. Boehler, Anna M. Raines, Roger A. Sunde

**Affiliations:** Department of Nutritional Sciences, University of Wisconsin-Madison, Madison, Wisconsin, United States of America; Auburn University, United States of America

## Abstract

Selenium (Se) is an element that in trace quantities is both essential in mammals but also toxic to bacteria, yeast, plants and animals, including *C. elegans*. Our previous studies showed that selenite was four times as toxic as selenate to *C.* elegans, but that deletion of thioredoxin reductase did not modulate Se toxicity. To characterize Se regulation of the full transcriptome, we conducted a microarray study in *C. elegans* cultured in axenic media supplemented with 0, 0.05, 0.1, 0.2, and 0.4 mM Se as selenite. *C. elegans* cultured in 0.2 and 0.4 mM Se displayed a significant delay in growth as compared to 0, 0.05, or 0.1 mM Se, indicating Se-induced toxicity, so worms were staged to mid-L4 larval stage for these studies. Relative to 0.1 mM Se treatment, culturing *C. elegans* at these Se concentrations resulted in 1.9, 9.7, 5.5, and 2.3%, respectively, of the transcriptome being altered by at least 2-fold. This toxicity altered the expression of 295 overlapping transcripts, which when filtered against gene sets for sulfur and cadmium toxicity, identified a dataset of 182 toxic-Se specific genes that were significantly enriched in functions related to oxidoreductase activity, and significantly depleted in genes related to structural components of collagen and the cuticle. Worms cultured in low Se (0 mM Se) exhibited no signs of deficiency, but low Se was accompanied by a transcriptional response of 59 genes changed ≥2-fold when compared to all other Se concentrations, perhaps due to decreases in Se-dependent TRXR-1 activity. Overall, these results suggest that Se toxicity in *C. elegans* causes an increase in ROS and stress responses, marked by increased expression of oxidoreductases and reduced expression of cuticle-associated genes, which together underlie the impaired growth observed in these studies.

## Introduction

Selenium (Se) is an element that in trace quantities is both essential in mammals but also toxic to bacteria, yeast, plants and animals, including *C. elegans*. In mammals, Se is essential because it is incorporated as selenocysteine (Sec) during translation into the peptide backbone of a small set of selenoproteins, at the position specified by an in-frame UGA codon and mediated by a unique tRNA [1]. Some bacteria and other prokaryotes also have retained this molecular machinery for specific incorporation of Se into protein [2]. In contrast, the toxicity of Se is far less understood. Concentrations >2 mM Se block growth in bacteria and yeast [3], [4]. In C. elegans, concentrations >2 mM Se are lethal both in long-term growth studies [5] as well as in short-term acute studies with adult C. elegans [6]. At the whole organism level, Se toxicity is observed in animals at greater than 20 times (>20X) the dietary Se requirement [7], [8]. In humans, adverse effects of Se also occur at roughly 20X the recommended dietary allowance (RDA) after long-term exposure [9]–[11], and acute toxicity has even occurred recently in the US due to misformulated health supplements that provided up to 740X the RDA [12]. Rodent cancer studies and human epidemiological and clinical trials, however, have shown that Se supplementation at levels 4-5X the RDA protects against a variety of cancers [13]–[15] and cardiovascular disease [16], whereas selenium supplementation is also associated with increased risk of cardiovascular disease and diabetes [17]–[19]. At the molecular level, Se toxicity is associated with alterations in thiol metabolism and redox state [20], with increased reactive oxygen species [21], and with alterations in protein synthesis [22], but the underlying molecular mechanisms important in Se toxicity and the metabolic pathways associated with Se toxicity are poorly understood.

The *C. elegans* genome encodes all the enzymes and the Sec-tRNA necessary for incorporation of Se as Sec into a single selenoprotein, thioredoxin reductase 1(TRXR-1) [5], [23]–[25]. We recently completed a study in *C. elegans* to investigate Se toxicity and the importance of thioredoxin reductase [5], a proposed key enzyme in Se metabolism [20], for protection against Se toxicity. L1 larvae were cultured for 12 days in axenic media supplemented with Se to prevent detoxification of Se by bacteria when used as a food source [3], [26]–[28]. In these long-term experiments, 0.2 mM Se as selenite reduced growth and delayed maturation to the adult stage, with an LC_50_ (Se concentration that results in 50% of plateau worm number) of 0.20 mM Se. We also found deletion of *trxr-1*, its cysteine homolog *trxr-2* or both (*trxr-1/2 −/−*) in *C. elegans* had no effect on susceptibility to Se toxicity, suggesting that multiple enzymes are involved in metabolizing toxic levels of Se [5]. Se toxicity has been demonstrated acutely over the course of 12 h in *C. elegans* with an LC_50_ of 3.47 mM Se as selenite [6]. To date, the transcriptional response of *C. elegans* to Se toxicity is yet to be characterized.

To better understand the effect of toxic Se as well as low Se on *C. elegans*, we conducted a microarray study in *C. elegans* cultured in axenic media supplemented with 0, 0.05, 0.1, 0.2, and 0.4 mM Se as selenite. Worms were cultured in these Se concentrations until they reached the mid-L4 stage [29] to avoid developmental differences due to slowed growth in high Se. Culturing worms in 0.2 and 0.4 mM Se to the L4-larval stage identified a set of 182 toxic-Se specific genes with expression changes ≥2-fold relative to 0.1 mM Se. Gene ontology (GO) analysis found that this dataset was significantly enriched in functions related to oxidoreductase activity, and significantly depleted in transcripts related to collagen and cuticle development. In worms cultured in low Se, functional clustering annotation identified genes associated with peptidase and methyltransferase activity.

## Materials and Methods

### Reagents

Molecular biology reagents were purchased from Promega (Madison, WI), Invitrogen (Carlsbad, CA) or Sigma (St. Louis, MO). All other chemicals were of molecular biology or reagent grade.

### General maintenance and growth conditions

The wild-type *C. elegans* strain used in all experiments is N2 Bristol (N2) obtained from the Caenorhabditis Genetic Center (CGC, Minneapolis, MN). Maintenance and growth of *C. elegans* is started initially under standard conditions on nematode growth media (NGM) agar plates with an *E. coli* OP50 lawn [30]. Following growth to gravid (egg-bearing) adults, *C. elegans* are bleached in 1.1% Clorox bleach (sodium hypochlorite)/0.55 M NaOH to release eggs, as described by Rao et al. [31]. Eggs are hatched overnight in M9 buffer to produce a synchronized population of growth-arrested L1 larvae. L1 *C. elegans* are then inoculated into cultures of CeHR-3 media, a defined liquid axenic developed by Iqbal Hamza’s group at the University of Maryland [31]. Our basal axenic media, containing 0.000125 mM Se, reduces TRXR activity to 80% of Se-supplemented levels [5], and thus is designated low Se.

### Staging *C. elegans* for RNA isolation

Synchronized L1 larvae prepared as described above were inoculated into 10 mL (100 L1/mL) of axenic media and grown for 6-7 days until they become gravid adults. The resulting gravid *C. elegans* were then treated with bleach/NaOH solution, eggs hatched overnight, and the resulting L1s were used to inoculate 10 mL (100 L1/mL) axenic cultures containing 0, 0.05, 0.1, 0.2, or 0.4 mM Se as sodium selenite (Sigma), with three independent replicates at each Se concentration. Worms were allowed to grow until ≥50% of the culture reached the mid-L4 stage in development, as denoted by the formation of a clear, crescent patch [29], which was assessed daily by microscopy (Amscope XSG-T110±10 MP camera) beginning at day 4, proceeding through day 12, including every 12 h as worms approached L4 stage.

### RNA isolation and microarray processing

Upon reaching the mid-L4 stage, worms were washed 3X in M9 buffer, pelleted to ∼150 µl, and total RNA was isolated with TRIzol Reagent (Invitrogen, Carlsbad, CA) following the manufacturer’s protocol. RNA concentration and purity were assessed using the NanoDrop ND-2000 UV-Vis Spectrophotometer (NanoDrop Technologies, Wilmington, DE), with A_280_/A_260_ and A_260_/A_230_ ratios between 1.8–2.1 being considered acceptable RNA. Total RNA from one replicate of each Se-treatment group was submitted to the Gene Expression Center (University of Wisconsin, Madison, WI). Briefly, RNA integrity was verified on the Agilent RNA6000 Pico Chip, and subjected to 3’ RNA labeling using the Ambion Message Amp Premier Kit (Life Technologies, Grand Island, NY). The resulting aRNA was purified, quantified, and fragmented, and then the aRNA (12 µg) was hybridized to Affymetrix Whole Genome *C. elegans* GeneChip arrays (GLP200, Affymetrix, Santa Clara, CA) according to the manufacturer’s protocols. These GeneChips contain 22,625 probe sets targeting 21,150 unique transcripts.

### Microarray analysis

The resulting data files for each microarray were analyzed with GeneSpring (Agilent Technologies, Santa Clara, CA, U.S.A.). Se treatments were initially compared relative to the 0.1 mM Se treatment to assess the whole range of transcript effects, from low Se to toxic Se. Affymetrix RMA signal values were viewed in scatter plots relative to the signal values for the 0.1 mM Se treatment. The data then was filtered for expression >100, normalized, and subjected to further analysis. The fold-change threshold was set at ≥2-fold and gene expression changes are described by fold-change. Unsupervised hierarchical clustering was performed on genes significantly altered by toxic Se (both 0.2 and 0.4 vs. 0.1 mM Se) and low Se (0 vs. 0.1 and 0.05 mM Se) using GeneSpring. GO analysis was used to identify processes significantly enriched in *C. elegans* in the resulting datasets. In addition, functional annotation clustering analysis was performed on the low-Se specific dataset using the Database for Annotation, Visualization and Integrated Discovery (DAVID) v6.7 (http://david.abcc.ncifcrf.gov/). Gene ontology terms and annotation clusters were considered significantly enriched if the adjusted *P*-value was <0.05. The microarray data discussed in this publication have been deposited in NCBI's Gene Expression Omnibus and are accessible through GEO Series accession number GSE54011 (http://www.ncbi.nlm.nih.gov/geo/query/acc.cgi?acc=GSE54011).

### qRT-PCR analysis

Selected genes found to be differentially expressed and/or significantly enriched in the microarray analysis were validated by qRT-PCR as previously described [5]. Triplicate cultures at each Se concentration were used in the analysis. Briefly, one µg of total RNA was reverse transcribed to cDNA using the RETROscript kit (AM1710, Ambion, Austin, TX, U.S.A.), following the manufacturer’s instructions. Primers to amplify cDNA targets were designed using Primer3 (http://biotools.umassmed.edu/bioapps/primer3_www.cgi) and when possible primer pairs spanned an exon/exon junction to ensure amplification of mRNA and not genomic DNA. The final real time reactions, containing reverse transcribed RNA, gene-specific forward and reverse primers, and 1X SybrGreen PCR Master Mix (#4309155, Applied Biosystems, Foster City, CA, U.S.A.), were performed in an ABI Prism 7000 Sequence Detection System (Applied Biosystems), with initial stages of 50°C for 2 min and 95°C for 10 min, followed by 50 cycles of 95°C for 15 sec and 60°C for 2 min. A dissociation curve was run for each plate to confirm production of a single product. The amplification efficiency for each gene was determined using the DART program. Relative abundance of each mRNA was calculated using the method of Pfaffl [32], accounting for gene specific efficiencies, and normalized to the mean of *act-1* and *eft-3* expression.

### Transcript filtering

Transcripts that were significantly regulated by toxic Se (in both 0.2 and 0.4 mM Se vs. 0.1 mM Se) were filtered to obtain an initial set of toxic-Se response transcripts. Transcripts in the this toxic-Se dataset were compared to transcripts altered by sulfur exposure in a recent *C. elegans* microarray [33] and to transcripts altered by cadmium toxicity in *C. elegans* [34]. Sulfur exposure microarray data was downloaded from the Gene Expression Omnibus at NCBI (GEO accession number GSE25199) and independently analyzed by comparing the *C. elegans* treatment group exposed to 50 ppm H_2_S for 48 h to control worms. Transcript expression changes in the cadmium toxicity dataset (GEO accession number GSE7535) were taken directly from Cui et al. [34], and compared to the toxic-Se dataset. The probe sets found to overlap between the initial toxic-Se dataset and these datasets were removed to identify a dataset of “toxic-Se specific” gene changes.

To identify low-Se specific gene changes, the set of transcripts changed ≥2-fold by 0 mM Se when compared to 0.1, 0.2, and 0.4 mM Se sets, was then refined by filtering against the set of transcripts that were changed ≥2-fold only by 0 mM Se and not by 0.1, 0.2, or 0.4 mM Se when compared to 0.05 mM Se to identify a “low-Se specific” dataset of transcripts.

To ensure that transcript changes were not due to developmental differences, the ≥2-fold changes for each Se concentration were compared with transcripts changed by the developmental transition of L4 larva to young adult stage in a study by Youngman et al. [35]. This dataset was downloaded from the Gene Expression Omnibus at NCBI (GEO accession number GSE 21784) and independently analyzed comparing day-6 adults to the late-L4 stage NB *C. elegans*.

### Statistical analysis

Differences in growth time and qRT-PCR fold-changes are presented as the mean ± SEM. All data were analyzed by a one-way analysis of variance (ANOVA) and a protected LSD t-test. Means were considered statistically different when *P*<0.05.

## Results

### *C. elegans* exposed to 0.2 and 0.4 mM Se have a significant lag in growth

These studies were conducted using synchronized L1 worms grown in low Se, axenic media supplemented with graded levels of Se as selenite to exclude bacterial metabolism of Se and bacterial selenoprotein synthesis [5]. To ensure the observed expression changes are Se-specific and not due to developmental differences, worms were grown until the mid-L4 stage of development, as denoted by the clear, crescent patch of the developing vulva [29]. On average, worms cultured axenically in 0.1, 0.2, and 0.4 mM Se required an additional 32, 52, and 52 h, respectively, as compared to 0 and 0.05 mM Se cultures, for ≥50% of the culture to reach L4 stage (**Table 1**). This is in agreement with our previous study where worms cultured in 0.2 mM Se had a significant delay in growth [5].

**Table 1:** Effect of media selenium on time for L1 larva to reach mid-L4 larval stage.

	Media Se (mM)				
	0	0.05	0.1	0.2	0.4
**Replicate**	**Time** (**h)**				
**A**	144	144	168	204	204
**B**	144	144	156	192	192
**C**	144	144	168	192	192
**Average**	144±0^A^	144±0^A^	164±4^A^	196±4^B^	196±4^B^

Time (hours) in CeHR-3 media supplemented with increasing Se concentrations (0–0.4 mM Se as selenite) to mid-L4 larval stage. A one-way ANOVA and protected LSD t-test were performed to analyze significant time differences across Se concentrations. Means with different letters were statistically different, *P*<0.05.

### Dietary selenium induces moderate transcript changes in *C. elegans*

**Figure 1:**
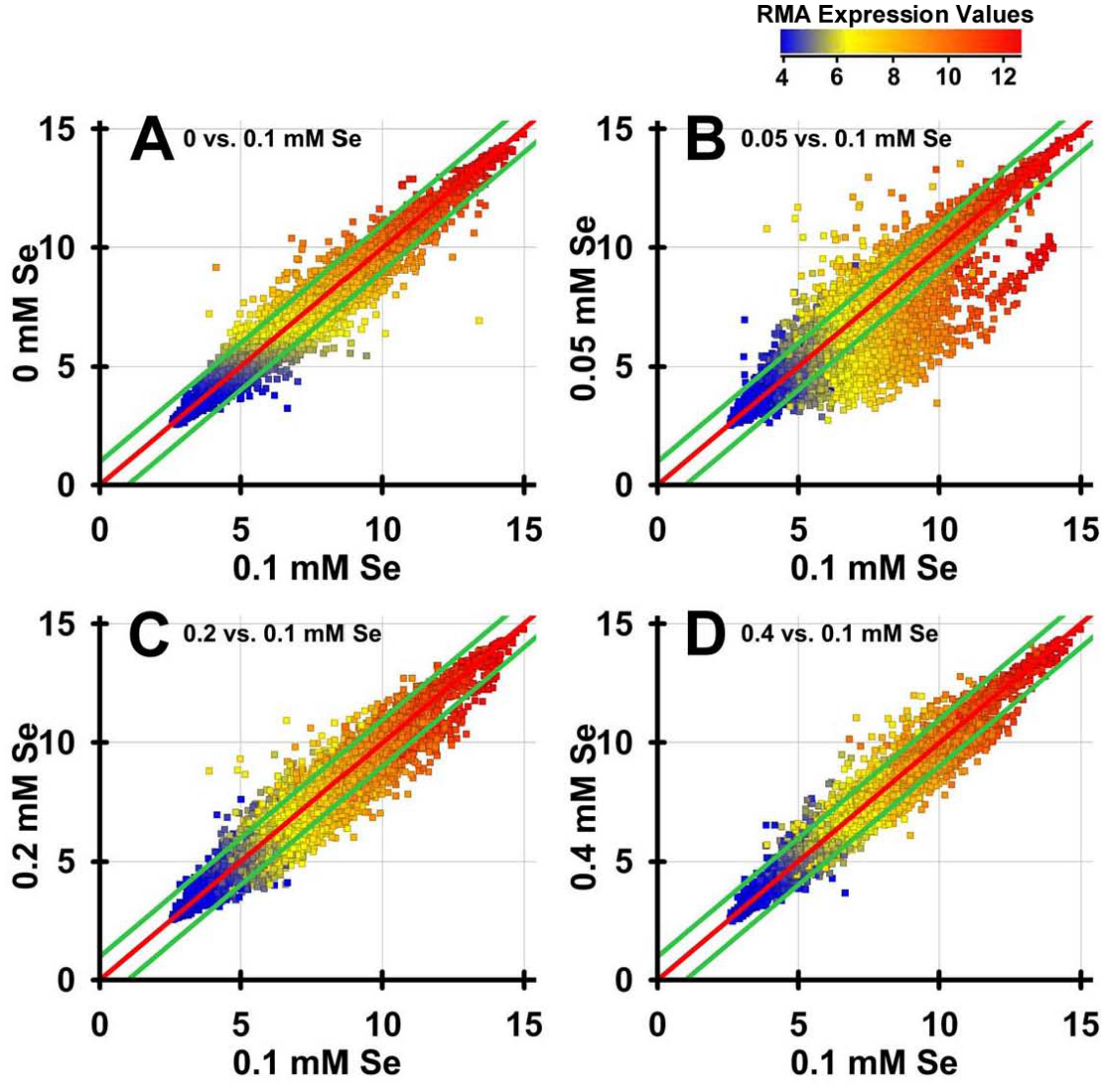
Effect of Se on the C. elegans transcriptome. Linear RMA expression values (non-normalized) from GeneSpring analysis of all 22,150 transcripts on the Affymetrix Whole Genome C. elegans GeneChip Array are plotted for 0 mM Se (A), 0.05 mM Se (B), 0.2 mM Se (C), and 0.4 mM Se (D) vs. expression values for 0.1 mM Se. Values on the x- and y-axis, and on the color legend, are the linear RMA expression data. The middle red line represents 1:1 expression, and the top and bottom green lines represent 2-fold up- and down-regulated expression, respectively. Each colored dot represents an individual transcript, with blue being a low abundance transcript and red being a high abundance transcript.

Transcript expression of *C. elegans* cultured in 0, 0.05, 0.2 and 0.4 mM Se as selenite was assessed using Affymetrix Whole Genome *C. elegans* GeneChip arrays (Affymetrix, Santa Clara, CA). To obtain a global picture of the effect of dietary Se treatment, a scatterplot of RMA expression of each non-normalized Se treatment was plotted against 0.1 mM Se data (**Fig. 1**). This figure shows relatively tight clustering of expression along the 1:1 line for all treatments. Overall, toxic Se resulted in more down-regulated transcripts as compared to up-regulated transcripts. In addition, there was an expanded scattering of transcripts changed ≥2-fold in the 0.05 mM Se vs. 0.1 mM Se (**Fig. 1B**), when compared to the scatter patterns for 0, 0.2 and 0.4 mM Se vs. 0.1 mM Se treatment (**Fig. 1A, 1C, 1D**), with the clear majority of the ≥2-fold transcripts in the 0.05 mM Se group down-regulated as compared to the 0.1 mM Se treatment.

**Figure 2:**
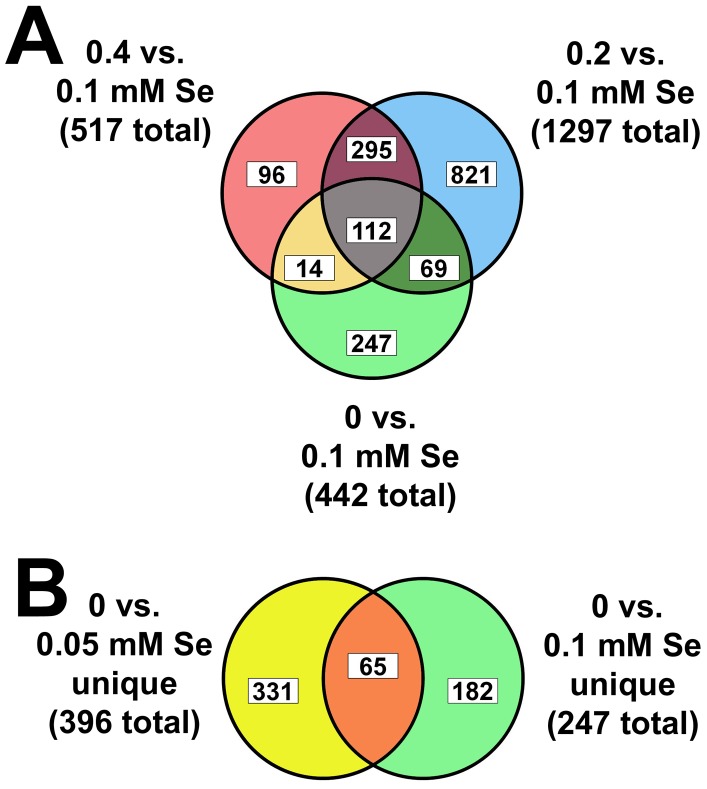
Identification of transcript datasets regulated by toxic Se and low Se. **A.** Plotted is the overlap of transcript sets regulated ≥2-fold relative to 0.1 mM Se treatment for 0, 0.2 and 0.4 mM Se treatments (442, 1297, 517 total transcripts in each set, respectively). The 295 overlapping transcripts for toxic Se (0.2 and 0.4 mM), and the 247 transcripts unique to low Se (0 mM vs 0.1 mM Se), were analyzed further for toxic Se and low Se changes. **B.** Plotted is the overlap of the 247 transcripts unique to low Se (0 mM) when compared to 0.1 mM Se (shown in A), and the 396 transcripts unique to low Se (0 mM) when compared to 0.05 mM Se, resulting in the “low Se specific” dataset of 65 transcripts changed ≥2-fold by 0 mM Se relative to both 0.05 and 0.1 mM Se.

This same general scatterplot pattern was observed when the treatments were compared to 0 mM Se data (data not shown), again with a large subset of 0.05 mM Se transcripts with lower expression, just as when compared to 0.1 mM Se. Conversely, when 0.05 mM Se was used as the comparison group, transcript clustering was expanded for all treatment groups (data not shown). On this basis, we selected the 0.1 mM Se dataset as the initial reference dataset for comparisons to identify transcripts with altered expression due to toxic or low Se. Relative to 0.1 mM Se, cultures supplemented with 0 mM Se had 442 probe sets with ≥2-fold altered expression (**Fig. 2A**). Supplementation with 0.05, 0.2 and 0.4 mM Se resulted in ≥2-fold expression of 2192, 1297, and 517 transcripts, respectively, indicating that 1.9%, 9.7%, 5.5%, and 2.3% of the transcriptome was altered by 0, 0.05, 0.2, and 0.4 mM Se relative to 0.1 mM Se.

### Transcript changes are primarily due to Se and not to developmental differences

These sets of transcripts that were changed ≥2-fold in L4 larva grown in 0, 0.05, 0.2, and 0.4 mM Se, relative to 0.1 mM Se, were compared against a set of developmental transcript changes associated with young adult vs. L4 larval stages [35]. Of the 6413 transcripts significantly changed ≥2-fold in young adults as compared to L4-larva, 93.8% (6017) were not changed ≥2-fold and in the same direction by any of the Se treatments. Key here, only 1.3% (85) were changed ≥2-fold and in the same direction by two or more of the Se treatments (**Fig. S1**). The lack of overlap shows that our staging process did not produce a significant population of adult worms in our treatment groups, and indicates that transcript changes seen in our study are primarily Se-specific.

### Toxic Se and low Se induce unique sets of transcript changes

The transcript expression changes induced by Se treatment in C. elegans are summarized quantitatively in **Fig. 2**. For transcripts altered ≥2-fold relative to 0.1 mM Se, 0.2 mM Se altered 1116 transcripts that did not overlap with 0 mM Se treatment, including 821 transcripts that were not altered ≥2-fold by any other treatment plus 295 transcripts that only overlapped with 0.4 mM Se treatment; 0.4 mM Se altered 391 transcripts that did not overlap with 0 mM Se treatment, including 96 transcripts that were not altered ≥2-fold by any other treatment plus the 295 transcripts overlapping only with 0.2 mM Se treatment (**Fig. 2A**). These 295 transcripts altered ≥2-fold by both 0.2 and 0.4 mM Se thus constitute an initial dataset of genes modulated by toxic Se.

Of 442 transcripts changed ≥2-fold by 0 mM Se, 247 of these transcripts did not overlap with transcripts in the 0.2 or 0.4 mM sets, as well as in the 0.1 mM Se set, thus identifying an initial low-Se dataset (**Fig. 2A**). When 0.05 mM Se was used as the comparison treatment, there were 396 transcripts uniquely changed ≥2-fold by 0 mM Se relative to 0.1, 0.2, and 0.4 mM Se (**Fig. S2**).

### Toxic-Se specific dataset

**Figure 3:**
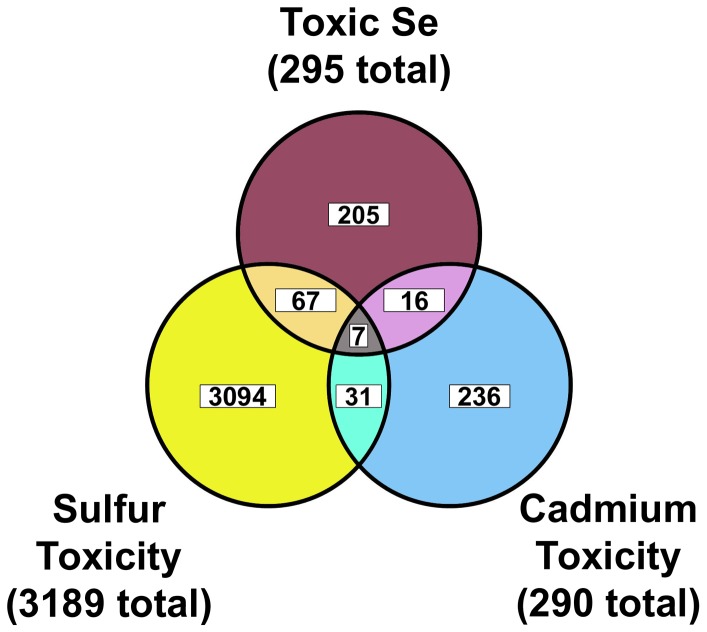
Identification of “toxic-Se specific” transcript dataset. The 295 overlapping toxic-Se transcripts in (0.2 and 0.4 vs. 0.1 mM Se) were filtered to remove the 67 transcripts that overlapped with transcripts changed ≥2-fold by sulfur toxicity [33], to remove the 16 transcripts that overlapped with transcripts changed ≥2-fold by cadmium toxicity [34], and to remove the 7 transcripts found in all sets, resulting in 205 transcripts. Removal of 23 duplicate transcripts resulted in the dataset of 182 “toxic-Se specific” genes.

The 295 overlapping transcripts in the 0.2 and 0.4 mM Se datasets could be due to a general toxicity response, as shown by the significantly decreased growth rates (**Table 1**). Thus we further refined this 295 toxic-Se dataset by filtering it against datasets from two published *C. elegans* microarray studies: a sulfur toxicity study, that examined 48 h exposure to 50 ppm sulfur [33], and a cadmium toxicity study, that examined transcript changes during exposure to 100 µM cadmium over 24 h [34]. Only 74 of the 295 transcripts in the initial toxic-Se set overlapped with the 3189 transcripts in the sulfur toxicity dataset, and only 23 of the 290 transcripts in the cadmium toxicity dataset overlapped with the initial toxic-Se dataset (**Fig. 3**). Only 7 transcripts were in all 3 datasets. The removal of the 90 overlapping transcripts, identified by overlap with the sulfur and cadmium toxicity studies, resulted in 205 transcripts. This dataset contained 23 duplicate transcripts or transcripts without any corresponding ID, thus yielding a total of 182 “toxic-Se specific genes” for analysis (**Table S1**).

### Se toxicity up-regulates oxidoreductases and down-regulates genes involved in collagen and cuticle development

**Figure 4:**
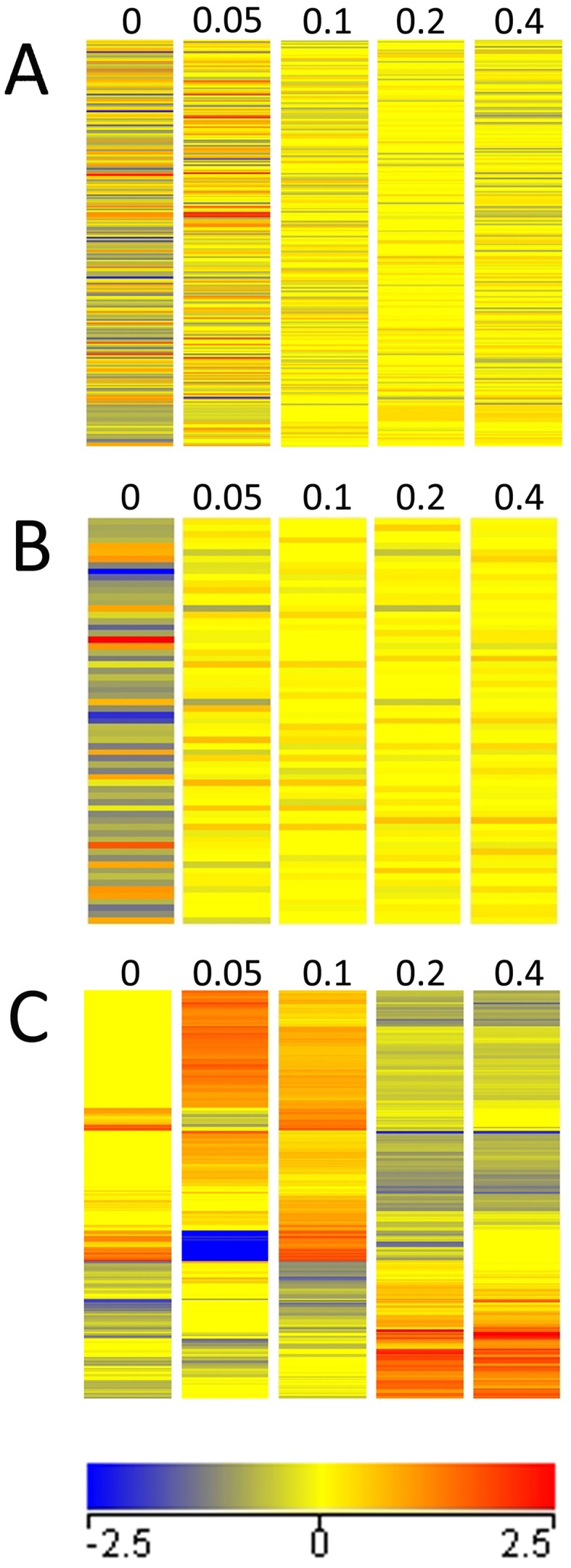
Hierarchical clustering of toxic-Se specific and low-Se specific datasets. Unsupervised hierarchical clustering of (A) 182 toxic-Se specific genes; (B) 247 low-Se transcripts; (C) 59 low-Se specific genes. Columns represent expression at 0, 0.05, 0.1, 0.2, and 0.4 mM Se. Gene expression is shown using a pseudocolor scale from −2.5 (blue) to +2.5 (red).

Unsupervised hierarchical clustering analysis of these 182 genes identified clusters of toxic-Se genes that were differentially up- or down-regulated by 0.2 and 0.4 mM Se when compared to 0, 0.05, and 0.1 mM Se (**Fig. 4A**). The majority of the 182 toxic-Se specific genes were significantly down-regulated, with 129 genes decreased ≥2-fold, versus 53 up-regulated genes. GO analysis of down-regulated genes in the toxic-Se specific dataset revealed 125 molecular functions, biological processes, and cellular components enriched by toxic Se (**Table S2**), including 7 distinct (10 listed) molecular functions (**Table 2**). Down-regulated functions were associated with structural components of collagen and the cuticle, peptidase activity, and DNA binding. GO analysis of up-regulated genes in the toxic-Se specific dataset revealed two GO terms enriched by toxic Se (**Table S2**), including oxidoreductase activity as the one molecular function (**Table 2**). This ontology analysis suggests Se toxicity in *C. elegans* induces up-regulation of genes required to maintain proper redox homeostasis, while down-regulating structural genes important for proper larval development.

**Table 2:** Molecular functions enriched by toxic selenium.

GO ACCESSION *	GO Term *	corrected p-value	Changed Genes †	Total Genes ‡	% Changed@
**Down-regulated**					
**GO:0042302**	structural constituent of cuticle	8.27E-12	161	17	10.6
**GO:0043565**	sequence-specific DNA binding	1.39E-10	527	26	4.9
**GO:0003677**	DNA binding	1.47E-05	927	26	2.8
**GO:0042329**	structural constituent of collagen and cuticulin-based cuticle	3.07E-05	9	4	66.7
**GO:0005198**	structural molecule activity	1.47E-04	504	17	3.4
**GO:0008233**	peptidase activity	3.22E-04	392	12	3.1
**GO:0003676**	nucleic acid binding	0.044	1525	26	1.7
**Up-regulated**					
**GO:0016491**	oxidoreductase activity	0.012	607	10	1.6

*Gene Ontology (GO) accession numbers and terms significantly enriched in the 182 Se-specific genes regulated by toxic Se.

† Number of genes involved in this process in the set of toxic-Se specific genes.

‡ Number of genes involved with this process in the GeneChip C. elegans Genome Array.

@Percent of genes changed in this process specific to toxic-Se compared to all genes in this process in the GeneChip C. elegans Genome Array.

### Low Se alters genes enriched for a single annotation cluster

The 247 transcripts in the initial low-Se dataset contained 25 duplicate transcripts yielding 222 corresponding genes for analysis. Hierarchical clustering revealed no distinct pattern of gene clustering as compared to other Se concentrations (**Fig. 4B**). This low-Se dataset was further refined by filtering against the 396 unique transcripts changed ≥2-fold by 0 mM Se when compared to 0.05 mM Se (**Fig. S2**), to identify the set of 65 transcripts changed ≥2-fold by low Se relative to both 0.05 and 0.1 mM Se (**Fig. 2B**). This set contained 6 duplicates, yielding 59 corresponding genes in a “low-Se specific gene” dataset (**Table S3**), with 46 genes decreased ≥2-fold, versus 13 up-regulated genes. Unsupervised hierarchical cluster analysis of these low-Se specific genes displayed a much more distinct pattern of gene clustering compared to the other Se concentrations (**Fig. 4C**). GO analysis of these 59 genes resulted in no enriched processes altered by low Se with *P*<0.05, but identified two genes in methyltransferase function with *P* = 0.22 (**Table 3**). These two genes, *R08E5.03* and *R08E5.1*, are the second- and third-most down-regulated in the low-Se specific dataset (**Table S3**). Using DAVID, functional annotation clustering of the low-Se dataset found a significant category of genes over-represented for peptidase activity (**Table 3**). In low-Se, four peptidase genes (*cpr-2, H19M22.3, T05E7.1, C44E12.1*) were down-regulated, and one peptidase gene (*K12H4.7*) was up-regulated.

**Table 3:** Molecular functions and gene clusters enriched in low selenium.

GeneSpring Gene Ontology Molecular Functions:					
GO ACCESSION *	Term	Changed Genes ‡	Total Genes †	Corrected p-value	% Changed@
**GO:0008175**	tRNA methyltransferase activity	2	12	0.225	16.7

DAVID Functional Annotation: Peptidase Activity Cluster; Enrichment Score = 1.10				
GO ACCESSION	Term	Gene Count	Fold Enrichment *	P-value *
**GO:0070011**	peptidase activity, acting on L-amino acid peptides	5	4.24	0.02
**GO:0008233**	peptidase activity	5	3.92	0.03
**GO:0006508**	proteolysis	5	3.20	0.06
**GO:0008237**	metallopeptidase activity	3	5.59	0.09

Analysis of the 59 overlapping gene products significantly altered by 0 mM Se as compared to 0.1 and 0.05 mM Se.

*Gene Ontology (GO) accession numbers and terms significantly enriched in the 59 low-Se specific genes.

† Number of genes involved in this process in the set of low-Se specific genes.

‡ Number of genes involved with this process in the GeneChip C. elegans Genome Array.

@Percent of genes changed in this process specific to low-Se compared to all genes in this process in the GeneChip C. elegans Genome Array.

### qRT-PCR confirmation of identified gene changes

Analysis of the full microarray dataset (expression >100) for transcripts altered ≥2-fold relative to 0 mM Se treatment identified 3076 transcripts. Representative transcript genes from this dataset, along with selected sulfur metabolism-related genes, were analyzed by qRT-PCR of total RNA from three independent cultures grown in each Se concentration (**Table 4** **, Fig. S3–S5**). For the seven oxidoreductases listed in **Table 4**, the fold changes determined by qRT-PCR analysis ranged from 1.32 to 4.70 at 0.2 mM Se, and 1.85 to 4.41 at 0.4 mM Se, thus confirming the microarray analysis. qRT-PCR analysis similarly confirmed that five sulfur metabolism-related genes, changed ≥2-fold as assessed by microarray analysis, were up-regulated by toxic Se (**Fig. S3, S4A–S4D**); this includes *gst-19* (in the glutathione-s-transferase family) and *cpr-2* (putative cysteine-type peptidase (**Fig. S5F**), which were elevated >35-fold by 0.2 or 0.4 mM Se treatment as assessed by either microarray or qRT-PCR. Similar up-regulation was found for two ER stress-related genes which were elevated >3-fold by 0.4 mM Se, as assessed by qRT-PCR. Additionally, two transporter gene transcripts were also up-regulated by toxic Se treatment as assessed by qRT-PCR (**Fig. S3E–S3H**). Lastly, qRT-PCR analysis of an additional five sulfur metabolism transcripts, which by microarray analysis were regulated <1.4-fold by 0.2 and 0.4 mM Se (including *trxr-1*, *trxr-2*, and *glrx-21*), confirmed the limited modulation of these transcripts by Se status (**Table 4** **, Fig. S4E–S4H**).

**Table 4:** qRT-PCR validation of gene expression changes, expressed as fold-change (FC) relative to 0 mM Se.

	Microarray †		qRT-PCR ‡	
Fold change	0.2 mM	0.4 mM	0.2 mM	0.4 mM
***Oxidoreductases***				
***sodh-1***	3.18	2.32	4.70±0.19***	3.05±0.32***
***hacd-1***	4.31	2.82	4.21±0.48**	3.39±0.37**
***cyp-14A1***	2.92	4.55	1.32±0.24	3.06±0.72
***cyp-33C7***	4.16	2.78	1.61±0.55	2.04±0.29*
***F55E10.6***	2.07	2.53	1.91±0.37*	2.53±0.33***
***fat-3***	2.31	2.03	3.38±0.63	4.41±0.42*
***dhs-9***	2.54	2.29	1.84±0.23*	1.85±0.16*
***Sulfur Metabolism***				
***cpr-2***	78.77	68.25	41.76±3.14*	36.1±3.49**
***gst-19***	41.54	36.2	33.05±1.37***	36.11±3.50**
***gst-16***	3.24	3.47	3.92±0.31***	5.24±1.34***
***cdo-1***	3.08	2.65	3.03±0.45*	2.45±0.31*
***sulp-8***	2.68	2.58	2.49±0.11***	2.60±0.42*
gsr-1@	1.07	1.04	1.48±0.11*	1.27±0.19
glrx-21@	−1.01	1.11	1.23±0.30*	1.28±0.29*
pst-1@	−1.03	−1.27	1.50±0.25***	1.39±0.21*
trxr-1@	1.34 $	1.24 $	2.04±0.94	2.90±0.38
trxr-2@	1.14 $	1.47 $	−1.14±0.17	1.47±0.18
***Collagen Related***				
***col-14***	−2.46	−2.14	−2.23±0.49***	−2.04±0.42***
***col-38***	−2.89	−2.13	−4.71±0.51**	−3.64±1.04*
***col-79***	−2.48	−2.12	−2.59±0.01***	−2.28±0.03***
***dpy-18***	−2.18	−2.25	−1.57±0.20***	−1.28±0.13*
***Molting Cycle/Development***				
***wrt-6***	−2.53	−2.25	−4.36±0.01**	−4.59±0.02**
grd-5@	−1.74	−1.68	−3.39±0.03***	−4.27±0.02***
***ER Stress***				
***hsp-16.1***	2.95	3.48	1.68±0.22***	3.04±0.36**
***hsp-16.48***	2.65	2.95	3.62±0.21***	3.47±0.51**
***Transporters***				
***aqp-1***	5.2	3.67	3.45±0.20***	2.77±0.41*
***glc-1***	3.29	3.04	3.06±1.18***	2.16±0.55
***Unknown Function***				
***F47C12.7***	−34.49	−27.67	−70.54±2.89**	−48.15±12.2**

Fold changes of selected gene transcripts, listed by ontology categories, expressed relative to expression in the 0 mM Se treatment group.

† Fold-change observed in the GeneChip C. elegans Genome Array data.

‡ Fold-change determined by qRT-PCR.

* *P*<0.05, **P<0.001, ***P<0.005, for qRT-PCR data determined on 3 replicated for each gene.

@Gene transcripts that did not have microarray fold changes >2 relative to the in the 0 mM Se treatment group.

$Fold change for *trxr-1* and *trxr-2* calculated using non-normalized expression values <100.

The microarray analysis indicated that collagen/cuticle and molting cycle/development-related transcripts were down-regulated by toxic Se. qRT-PCR confirmed this down regulation for 6 genes (**Table 4** **, Fig. S5A–S5D**). Most interestingly, *F47C12.7* transcript levels, for a gene of unknown function, were down-regulated by 0 mM Se 27 to 70-fold relative to 0.05–0.4 mM Se (**Fig. S5E,** **Table 4**) as shown by microarray and by qRT-PCR. Collectively, qRT-PCR analyses validated the responses in *C. elegans* due to toxic Se and low Se that were observed by microarray analysis.

## Discussion

*C. elegans* in these studies were cultured in axenic media to better control the level of supplemented Se. Using this model, selenite at 0.2 mM or greater is toxic as demonstrated by delayed growth. In our previous study, we showed that worms cultured in 0.2 mM Se delayed growth but not reproduction, that worms cultured in 1 mM Se failed to develop to adults, and that worms cultured in 2 mM Se showed no development beyond the L1 larval stage [5]. In the present study, using C. elegans grown in synchronous cultures in 0.2 and 0.4 mM Se until the mid-L4 stage of development, we now have identified a set of 182 toxic-Se specific genes that were changed ≥2-fold relative to 0.1 mM Se and that were distinct from genes changed by sulfur and cadmium toxicity. The basal axenic media, which contains 0.000125 mM Se and which reduces TRXR activity to 80% of Se-supplemented levels [5], is thus designated “low Se.” This media appears to not be Se deficient because there is no associated growth or physical phenotype. These studies, however, have identified a set of 59 low-Se specific genes that provide a transcription-phenotype for Se-deficiency in *C. elegans*.

This is the first whole genome transcriptome study in *C. elegans* that examined the effects of Se status across the full range from potential Se-deficiency to Se toxicity. We found a moderate transcriptional response in *C. elegans* by altering the level of Se in the axenic media. With 22,625 probe sets, 1.9 to 9.7% of all transcripts were significantly altered ≥2-fold by 0, 0.05, 0.2, or 0.4 mM Se, as compared to 0.1 mM Se. When filtered against sulfur and cadmium toxicity, the initial toxic-Se set of 295 transcripts was refined to a dataset of 182 “toxic-Se specific genes.” Analysis of these toxic-Se specific genes identified enriched functions related to oxidoreductase activity, all being up-regulated, and enriched functions related to collagen and cuticle development that were down-regulated. Analysis of the low-Se (0 mM Se) dataset, revealed 59 “low-Se specific genes,” which were enriched in genes related to peptidase and methyltransferase activity.

Because growth was delayed by 0.2 and 0.4 mM Se in this study and by 0.2 to 5 mM Se in our previous study [5], we harvested worms at the same developmental stage to establish a true Se-specific transcriptional response. To verify this staging, the Se treatment datasets were compared to transcript changes that were reported in young adult vs. L4-stage larvae in a *C. elegans* aging study [35]. Only 1.3% of the 6413 young adult transcripts were changed ≥2-fold and in the same direction by two or more of the Se treatments (**Fig. S1**), clearly indicating that Se treatment rather than developmental stage was the predominant factor responsible for the observed transcript changes.

The fold changes found using microarray analysis were confirmed by qRT-PCR on RNA from three independent cultures at each Se concentrations (**Table 4**). For genes up-regulated by toxic Se (**Fig. S3, Fig. S4A–S4D**), this included *cpr-2* and *gst-19*, which were the most up-regulated genes relative to 0 mM Se treatment. This also included seven oxidoreductases plus three additional sulfur metabolism–related genes, two ER stress genes, and two transporter genes. In addition, we conducted qRT-PCR analysis for five sulfur-metabolism related genes that were not shown by microarray analysis to be regulated ≥2-fold by Se treatment; qRT-PCR confirmed that Se status had only modest and variable impact on transcript levels of these marginally-regulated genes (**Fig. S4E–S4G**). For genes up-regulated by low Se (shown as down-regulated by toxic Se in **Table 4**), qRT-PCR confirmed the changes found by microarray analysis, including for six collagen/cuticle and molting-related genes, and including F47C12.7 (**Fig. S5A–S5E**).

### Toxic-Se specific genes

The present study identified a toxic-Se specific set of 182 regulated genes that were altered in both the 0.2 and 0.4 mM Se datasets, and that did not overlap with sulfur and cadmium toxicity datasets. GO analysis identified significant up-regulation of 10 genes enriched for oxidoreductase function. This up-regulation strongly suggests that Se toxicity causes an increase in ROS. This was accompanied by an increase in expression of heat-shock protein genes, further indicating that Se-induced stress in *C. elegans* is associated with Se toxicity. In agreement, a microarray study with *S. cerevisiae* cultured in 1 mM Se as selenite reported significant up-regulation of oxidoreductase and protein degradation genes, indicative of a Se-induced stress response [36]. In our study, several of the up-regulated oxidoreductase genes in the toxic-Se specific gene set likely play roles in sulfur metabolism, including glutathione S-transferases and sulfur transferases (*gst-20, mpst-3, F01D5.10, F47B8.3, cysl-2*) (**Table 4** **, Table S1**). Multiple *gst* genes (*gst-4*, *gst-5, gst-16, gst-30)* were not included in the high-Se specific dataset because they are also present in the sulfur- and or cadmium-toxicity datasets. In yeast in a study investigating Se-resistance, a moderate selenite resistance was seen when the yeast glutathione reductase gene, glr1 [4], was overexpressed. Thus the results of the present study and other similar studies clearly link Se toxicity with responses that maintain proper oxidative capacity.

A sulfur transport gene, *sulp-8* (**Table 4**), and the putative transporter, *mpst-3*, with thiosulfate sulfurtransferase activity, were up-regulated, further emphasizing the overlap of Se toxicity with sulfur metabolism. Increased expression of other transporters such as aquaporin 1 (*aqp-1*) and glutamate-like channel 1 (*glc-1*) in the Se toxicity specific dataset (**Table 4**), suggests that there may be additional transport or excretion changes due to high Se. Recently in yeast, a monocarboxylate transporter, Jen1p, was identified as a high affinity selenite transporter, which when knocked-out, increased resistance to selenite [37]. This role of Jen1p in yeast provides a clear example where Se can “hitch-hike” along portions of pathways of more abundant metabolites rather than depending on Se-specific pathways. The monocarboxylate transporter genes in our *C. elegans* study, however, were unchanged by Se toxicity. The up-regulation of *aqp-1* and *glc-1* raises the question about the role of these transporters in Se metabolism.

In addition to the up-regulation of oxidoreductases genes, functional analysis of the toxic-Se specific genes revealed 17 down-regulated genes related to collagen and cuticle development (**Table 2**); six of these changes were confirmed by qRT-PCR (**Table 4**). In *C. elegans*, molting occurs at the end of each of the four larval stages and is marked by the shedding and synthesis of a new cuticle [38], [39]. A substantial portion of the cuticle is made up of collagens that are highly cross-linked, in part through disulfide bonds linking intermolecular cysteine residues [38], [39]. Stenvall et al. [40], recently showed that deletion of *trxr-1* or *trxr-2* or both was without effect in *C.elegans*, as was depletion of glutathione reductase (gsr-1) by RNAi, but with the combined loss of *trxr-1* and *gsr-1*, disulfide groups in the cuticle remain oxidized and result in a molting defect that severely impacts development. Our microarray analysis, however, only found <2-fold changes for *gsr-1* with toxic or low Se treatment, and there were also <2-fold changes for *bli-3*, *mlt-7*, and *pdi-2* (data not shown) which have been reported to be important in molting [41], [42]. Morgan et al. [6] further showed that Se toxicity in *C. elegans* increases circulating ROS, that deletion of *glrx-21* makes C. elegans hypersensitive to Se, and that loss of *glrx-21* eliminates the protective effect of exogenous glutathione [6]. Our study does suggest that Se toxicity may increase ROS in *C. elegans* which in turn results in derangement of cuticle disulfides, delaying growth. Considering these previous studies [6], [40], however, we were surprised to see <2-fold changes for *gsr-1* and *glrx-21* with toxic or low Se treatment, indicating that other key pathways and enzymes (Table 2) respond to changes in Se status.

Several mammalian studies have indicated an effect of Se toxicity on collagen [9], [43]–[46]. Chronic supplementation with excess Se results in collagen accumulation in collagenous tissues such as the skin, nails and hair [9]–[11], [45]–[47]. Similar to our results, several collagen genes were reported significantly down-regulated in human prostate cancer cell lines (LNCaP) supplemented with Se as Se-methylselenocysteine [43]. A study from our group in rats supplemented with up to 50X the requirement (5 µg Se/g diet), found extracellular matrix genes were particularly affected by Se toxicity, including 4 collagen genes, although the direction of this effect was up-regulation [44], further suggesting a link between Se toxicity and collagen metabolism.

In addition to enrichment of functions related to oxidoreductase activity and collagen/cuticle function, toxic Se significantly enriched functions related to DNA binding (**Table 2**). Down-regulated transcripts with this putative function include five histone genes (*his-1*, *his-11*, *his-12*, *his-20*, *his-48*), with wide homologies ranging from mammals to plants, and a leucine-zipper gene, *zip-11*, with narrow homology in seven species (2480}. This enrichment strongly suggests that a distinct modulation of nucleosome assembly and of sequence-specific DNA binding results from toxic Se treatment in *C. elegans*.

### Low-Se specific genes

Initial analysis identified a set of 222 genes (247 transcripts) changed by low Se (0 mM Se) vs. 0.1, 0.2 and 0.4 mM Se (**Fig. 2A**). Functional analysis of the 129 down-regulated genes in this dataset found no enriched processes. Analysis of the 93 up-regulated genes in this initial low-Se dataset, however, identified a significant up-regulation of gene expression related to lipid modification, specifically lipid glycosylation (data not shown). Further refinement of this low-Se data, however, by removing transcripts that were not also changed ≥2-fold by 0 vs. 0.05 mM Se treatment (**Fig. 2B**), resulted in a set of 59 “low-Se specific genes. *F47C12.7* is the gene most up-regulated by low Se and at the top of the low-Se specific dataset (**Table S3**). *F47C12.7* encodes an 185 amino acid protein of unknown function; computational phylogeny has only identified one additional homolog in *C. elegans*, plus one homolog in *C. briggsae* [48]. This gene is not present in the cadmium- and sulfur toxicity datasets, suggesting it is may be unique to low Se treatment in C. elegans.

The three genes most down-regulated by low Se are *cpr-2*, *R08E5.03* and *R08E5.1*. *cpr-2* (a putative cysteine-type peptidase) was down-regulated >20-fold relative to 0.05 and 0.1 mM Se; levels of *cpr-2* in 0.4 mM and 0.2 mM Se were altered <2-fold relative to 0.1 or 0.05 mM Se, and so was not included in the list of toxic-Se specific genes. These three genes might be good biomarkers for low Se status (see below).

GO analysis of the low-Se specific gene dataset identified two marginally enriched genes (*R08E5.03* and *R08E5.1*, *P* = 0.22) that are down-regulated by low Se. These genes are putative guanine-N7-methyltransferases, suggesting that tRNA maturation or mRNA capping is altered in low Se [49], [50]. Functional clustering analysis using DAVID, however, resulted in a cluster of genes down-regulated for peptidase activity. In our previous *C. elegans* study, 0 mM Se moderately reduced total TRXR activity, did not alter mRNA levels for the lone *C. elegans* selenoprotein, *trxr-1*, but increased the expression of its cysteine homolog, *trxr-2*. There is no Se-deficient growth-impairment phenotype in *C. elegans* grown in the low-Se media used in these studies, and deletion of *trxr-1*, *trxr-2* or both in *C. elegans* is without phenotype [5]. The 59 genes in the low-Se specific dataset, however, do provide a transcription-phenotype for Se-deficiency in *C. elegans*. Hypothetically, these changes might be modulated by reduced TRXR-1 activity in low-Se media. Microarray analysis of altered transcripts in *trxr-1* KO strains compared to 0 mM Se transcripts could determine if similar patterns arise with genetic Se deficiency versus nutritional Se deficiency in *C. elegans*.

### Mid-range Se effect

The expanded set of ≥2-fold changes with 0.05 mM Se, with 2192 transcripts altered ≥2-fold when compared to 0.1 mM Se, was unexpected. In our previous study, 0.05 mM Se was in the growth plateau between 0 and 0.1 mM Se in Se dose response curves, and 0.05 mM Se was sufficient to provide Se for maximal TRXR activity [5]. Thus we expected 0.05 mM Se transcript expression would be similar to that in 0 and 0.1 mM Se. As seen in **Fig. 1B**, this was not the case. The large transcript expansion of down-regulated genes at 0.05 mM Se relative to 0.1 and 0 mM Se suggests, perhaps, that 0.05 mM Se represents a Se-neutral concentration providing sufficient Se for Se-dependent TRXR-1 synthesis, and yet at a Se level insufficient to tax metabolism related to Se toxicity.

### Comparison of Se regulation with other species

Global effects of Se supplementation on the transcriptome in other species are varied. In *S. cerevisiae*, exposure to 1 mM Se as selenite resulted in a large transcriptional response, where approximately 30% of the open reading frames displayed significant gene changes related to oxidative stress and protein degradation [36]. This large response is most likely a general toxicity response as *S. cerevisiae* do not contain selenoproteins [51]. Studies in cancer cell lines, in contrast, have not identified a consistent set of gene changes associated with Se supplementation as high as 22 µM Se [43], [52]–[55]. Several rodent microarray studies with supplementation up to 1.0 µg Se/g diet reported 14-242 genes were altered, but the only consistently regulated genes in these studies were the selenoproteins [56]–[58]. Importantly, the design of these studies directly compared Se supplementation groups to Se-deficient groups, suggesting these reported changes were most likely due to Se-deficiency. A rat study from our group compared Se adequate rats (0.24 µg Se/g diet) to rats supplemented with Se as selenite up to 50X the requirement (5.0 µg Se/g diet). Only small transcriptional changes (<10 transcripts) were observed with up to 20X the requirement (2.0 µg Se/g diet), but at 5.0 µg Se/g diet, 1193 transcripts (4% of the genome) were significantly altered; these changes included genes enriched for processes of cell movement/morphogenesis, extracellular matrix, and development/angiogenesis [44]. Filtering of these transcripts against datasets for general toxicity and calorie restriction was used to make the gene set more Se-specific, but the variety of enriched biological processes suggests that a number of the alterations in these processes are downstream of direct effects of Se toxicity [44].

Previously, we reported that Se had a negligible effect on *trxr-1* transcript levels in *C. elegans* over the range from 0 to 0.25 mM Se [5]. *Trxr-1* transcript expression in the present microarray study was so low it did not reach the threshold of 100 for analysis. When non-normalized RMA values were analyzed, there were negligible fold changes in *trxr-1* mRNA over the range of 0 to 0.4 mM Se. When assessed by qRT-PCR, Ct values for *trxr-1* (and *trxr-2*) averaged 10-fold higher than the reference transcripts and were not significantly different (**Table 4**). In rats, in contrast, transcripts for 7 of 24 liver selenoproteins are significantly down-regulated by Se deficiency, including Gpx1, Sepw1, Selh, and Selk, which are down-regulated 8.2, 5.4, 3.2 and 2.4-fold, respectively, relative to Se-adequate levels [59]. Down-regulation of certain selenoprotein mRNA in mammals in Se deficiency is thought to regulate the redirection of limited Se to important selenoproteins [60], whereas in *C. elegans* with only 1 selenoprotein, there is not the need for a hierarchy in regulation.

### Unique and overlapping toxic-nutrient gene sets

**Fig. 3** clearly illustrates that these studies identified a set genes responding to toxic Se that is distinct from genes responding to toxic sulfur or cadmium, but also identified common sets of overlapping genes. For instance, the glutathione transferase, *gst-19*, is up-regulated ≥2-fold by toxic Se relative to 0.1 mM Se, but is also down-regulated ≥2-fold by low Se relative to 0.1 mM Se. *gst-19* is also present in both the cadmium- and sulfur toxicity datasets, and thus is present as one of the 112 common overlapping transcripts and not included as a toxic-Se specific gene. In fact, a total of nine and six *gst* genes are present in the sulfur and cadmium toxicity datasets, respectively. In contrast, *col-14*, *col-79*, *wrt-6*, *aqp-1*, and *hsp-16.1* were all unique to the toxic-Se specific dataset. Similarly, the unique up-regulation of *F47C12.7* combined with down-regulation of *R08E5.03* and *R08E5.1* in low Se have potential as biomarkers of low Se status. Thus the identification of the 182 toxic-Se and 59 low-Se specific genes in our study, including genes such as *F47C12.7*, suggests that unique subsets of gene transcripts specific for a given nutritional or toxic condition, such as the high-Se or low-Se specific gene sets, could be used to develop biomarker panels to assess status, requirements, and toxicity [61].

The results from this study show that *C. elegans* cultured in 0.2 and 0.4 mM Se have a significant delay in growth that is accompanied by expression changes of 182 toxic-Se specific genes. Se toxicity results in significant up-regulation of genes with oxidoreductase function and down-regulation of genes with functions related to collagen and cuticle formation. Low Se results in no associated growth phenotype, but is accompanied by a low-Se transcriptional response, perhaps due to changes in Se-dependent TRXR-1 activity. Overall, these results suggest that Se toxicity in *C. elegans* causes an increase in ROS and stress responses, marked by increased expression of oxidoreductases and reduced expression of cuticle-associated genes, which together underlie the impaired growth observed in these studies. In addition, these identified genes sets offer clues to how this organism responds to toxic as well as low Se, and may help to identify better biomarkers of Se status.

## Supporting Information

Figure S1 Se-specific versus developmental changes. The 442, 2192, 1297, and 517 transcript sets that were changed ≥2-fold by 0, 0.05, 0.2 and 0.4 mM Se, respectively, vs. 0.1 mM Se, were filtered against the set of 6413 transcripts significantly changed ≥2-fold in young adults as compared to L4-larva *C. elegans* [35]. Shown are the numbers of overlapping transcripts between these two studies that were changed ≥2-fold and in the same direction. Out of the 6413 young adult transcripts, 6017 were not changed ≥2-fold and in the same direction by any of the Se treatments. Only 85 overlapping transcripts were changed in this manner by 2 or more Se treatments (1, 2, and 82 transcripts for 4, 3, and 2 Se treatments, respectively), and 311 overlapping transcripts were changed by just one Se treatment (31, 256, 16 and 8 transcripts for 0, 0.05, 0.2 and 0.4 mM Se, respectively).
(TIF)

Figure S2 Identification of transcript datasets uniquely regulated by low Se relative to 0.05 mM Se. **A.** Plotted is the overlap of transcript sets changed ≥2-fold relative to 0.05 mM Se treatment for 0, 0.1, 0.2 and 0.4 mM Se treatments (2344, 2192, 1543, 2688 total transcripts in each set, respectively). The result is the set of 396 transcripts unique to low Se (0 mM) when compared to 0.05 mM Se.
(TIF)

Figure S3 Effect of Se on relative transcript levels of oxidoreductase activity (A–D), ER stress (**E–F**) and transporter (**G–H**) genes. Transcript levels are shown as fold change (FC) relative to levels in the 0 mM Se group (FC  = 1 or −1), as determined by microarray (•) and qRT-PCR (▪) on total RNA. Microarray values are FC of RMA generated expression (>100) values. qRT-PCR values are FC of mean ± SEM (n = 3) values, expressed relative to the mean of *act-1* and *eft-3* levels in each sample, from independent triplicate cultures grown in 0 to 0.4 mM Se. Shown are: oxidoreductase activity genes (A) *sodh-1*, (B) *hacd-1*, (C) *cyp-33C7*, (D) *fat-3*; ER stress genes (E) *wrt-6*, (F) *grd-5* genes; transporter genes (G) *aqp-1*, (H) *glc-1*.
(TIF)

Figure S4 Effect of Se on relative transcript levels of highly-regulated (A–D), and marginally-regulated (E-H) oxidoreductase/sulfur metabolism genes. Transcript levels are shown as fold change (FC) relative to levels in the 0 mM Se group (FC  = 1 or −1), as determined by microarray (•) and qRT-PCR (▪) on total RNA. Microarray values are FC of RMA generated expression (>100) values. qRT-PCR values are FC of mean ± SEM (n = 3) values, expressed relative to the mean of *act-1* and *eft-3* levels in each sample, from independent triplicate cultures grown in 0 to 0.4 mM Se. Shown are: highly-regulated genes (A) *gst-19*, (B) *gst-16*, (C) *cdo-1*, (D) *sulp-8*; marginally-regulated genes (E) *gsr-1*, (F) *glrx-21*, (G) *trxr-1*, (H) *trxr-2* genes. Fold change for *trxr-1* and *trxr-2* calculated using non-normalized expression values <100.
(TIF)

Figure S5 Effect of Se on relative transcript levels of collagen related (A–B), molting cycle related (C–D), and low-Se regulated (E–F) genes. Transcript levels are shown as fold change (FC) relative to levels in the 0 mM Se group (FC  = 1 or −1), as determined by microarray (•) and qRT-PCR (▪) on total RNA. Microarray values are FC of RMA generated expression (>100) values. qRT-PCR values are FC of mean ± SEM (n = 3) values, expressed relative to the mean of *act-1* and *eft-3* levels in each sample, from independent triplicate cultures grown in 0 to 0.4 mM Se. Shown are: collagen related genes (A) *col-14*, (B) *col-38*; molting cycle related genes (C) *wrt-6*, (D) *grd-5*; low-Se regulated genes (E) *F47C12.7*, (F) *cpr-2.*
(TIF)

Table S1 Set of Toxic-Se Specific Genes.
(DOC)

Table S2 Gene Ontology terms enriched by toxic selenium.
(DOC)

Table S3 Set of Low-Se Specific Genes.
(DOT)
