# Supplementary figures and images for: Toxic-Selenium and Low-Selenium Transcriptomes in Caenorhabditis elegans: Toxic Selenium Up-Regulates Oxidoreductase and Down-Regulates Cuticle-Associated Genes
Source: PLoS One. 2014 Jun 27;9(6):e101408. doi: 10.1371/journal.pone.0101408 (PMC4074201; doi:10.1371/journal.pone.0101408)

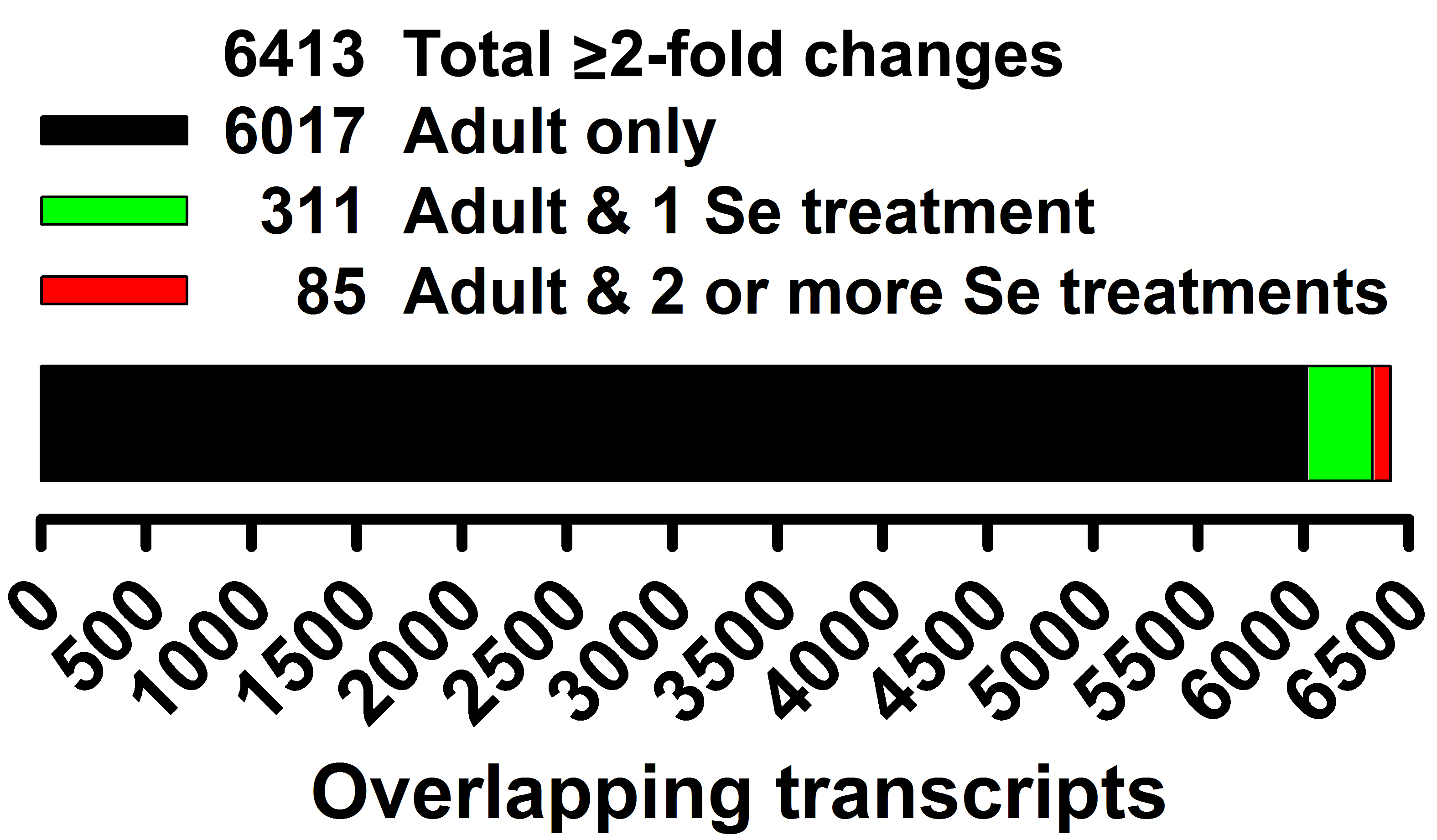

Supplement: Figure S1 — Se-specific versus developmental changes. The 442, 2192, 1297, and 517 transcript sets that were changed ≥2-fold by 0, 0.05, 0.2 and 0.4 mM Se, respectively, vs. 0.1 mM Se, were filtered against the set of 6413 transcripts significantly changed ≥2-fold in young adults as compared to L4-larva C. elegans [35]. Shown are the numbers of overlapping transcripts between these two studies that were changed ≥2-fold and in the same direction. Out of the 6413 young adult transcripts, 6017 were not changed ≥2-fold and in the same direction by any of the Se treatments. Only 85 overlapping transcripts were changed in this manner by 2 or more Se treatments (1, 2, and 82 transcripts for 4, 3, and 2 Se treatments, respectively), and 311 overlapping transcripts were changed by just one Se treatment (31, 256, 16 and 8 transcripts for 0, 0.05, 0.2 and 0.4 mM Se, respectively). (TIF) [file pone.0101408.s001.tif]

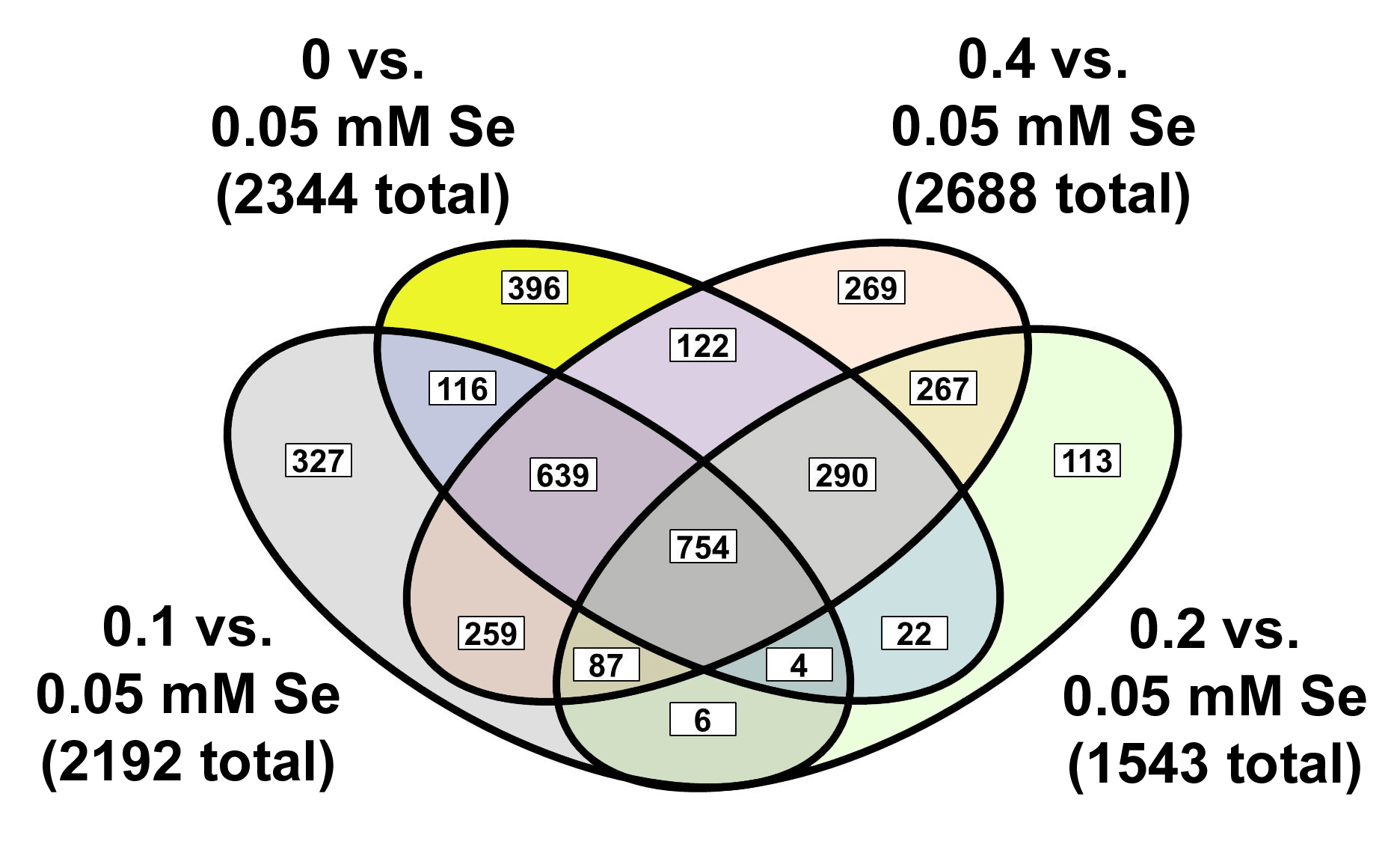

Supplement: Figure S2 — Identification of transcript datasets uniquely regulated by low Se relative to 0.05 mM Se. A. Plotted is the overlap of transcript sets changed ≥2-fold relative to 0.05 mM Se treatment for 0, 0.1, 0.2 and 0.4 mM Se treatments (2344, 2192, 1543, 2688 total transcripts in each set, respectively). The result is the set of 396 transcripts unique to low Se (0 mM) when compared to 0.05 mM Se. (TIF) [file pone.0101408.s002.tif]

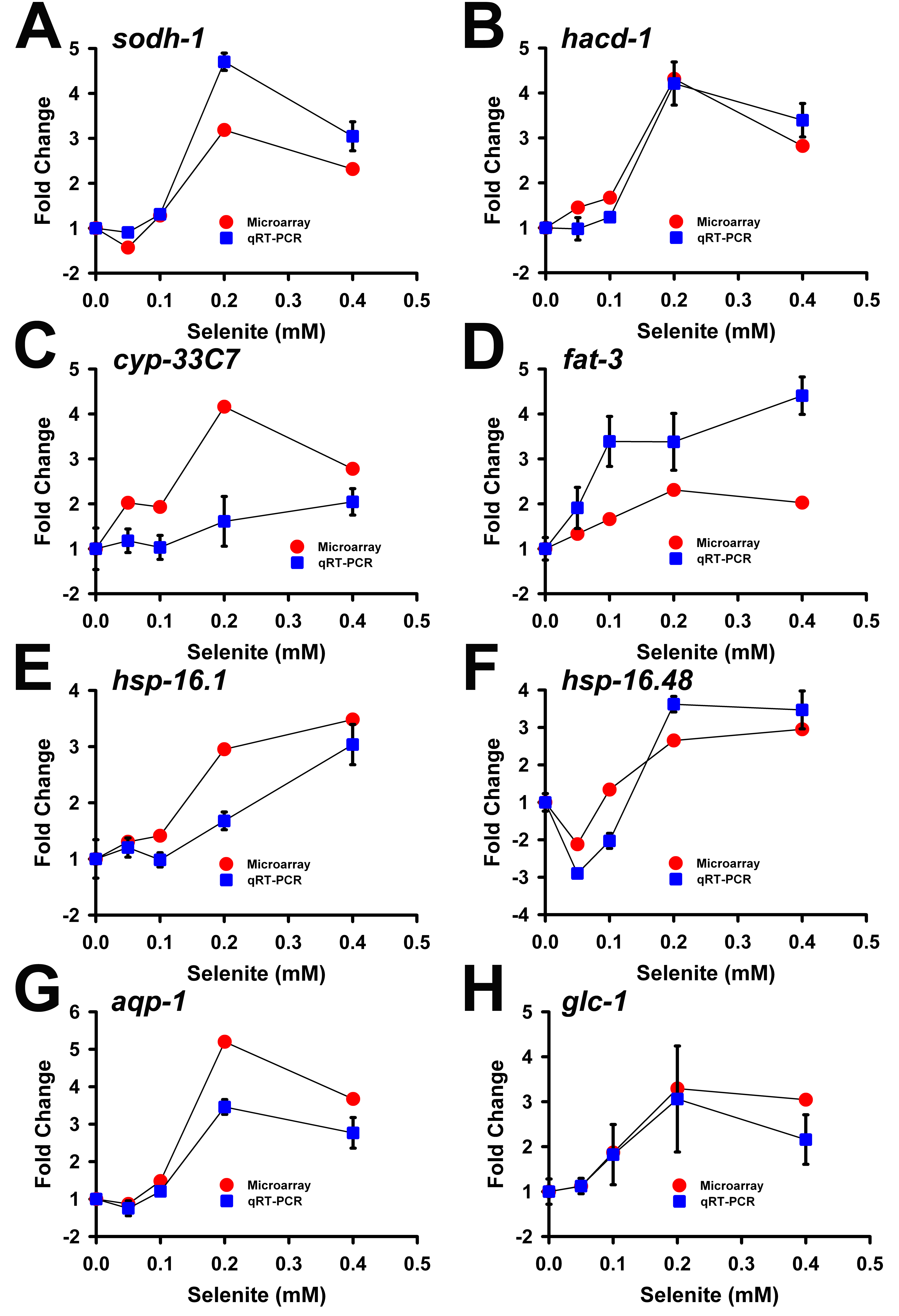

Supplement: Figure S3 — Effect of Se on relative transcript levels of oxidoreductase activity (A–D), ER stress (E–F) and transporter (G–H) genes. Transcript levels are shown as fold change (FC) relative to levels in the 0 mM Se group (FC = 1 or −1), as determined by microarray (•) and qRT-PCR (▪) on total RNA. Microarray values are FC of RMA generated expression (>100) values. qRT-PCR values are FC of mean ± SEM (n = 3) values, expressed relative to the mean of act-1 and eft-3 levels in each sample, from independent triplicate cultures grown in 0 to 0.4 mM Se. Shown are: oxidoreductase activity genes (A) sodh-1, (B) hacd-1, (C) cyp-33C7, (D) fat-3; ER stress genes (E) wrt-6, (F) grd-5 genes; transporter genes (G) aqp-1, (H) glc-1. (TIF) [file pone.0101408.s003.tif]

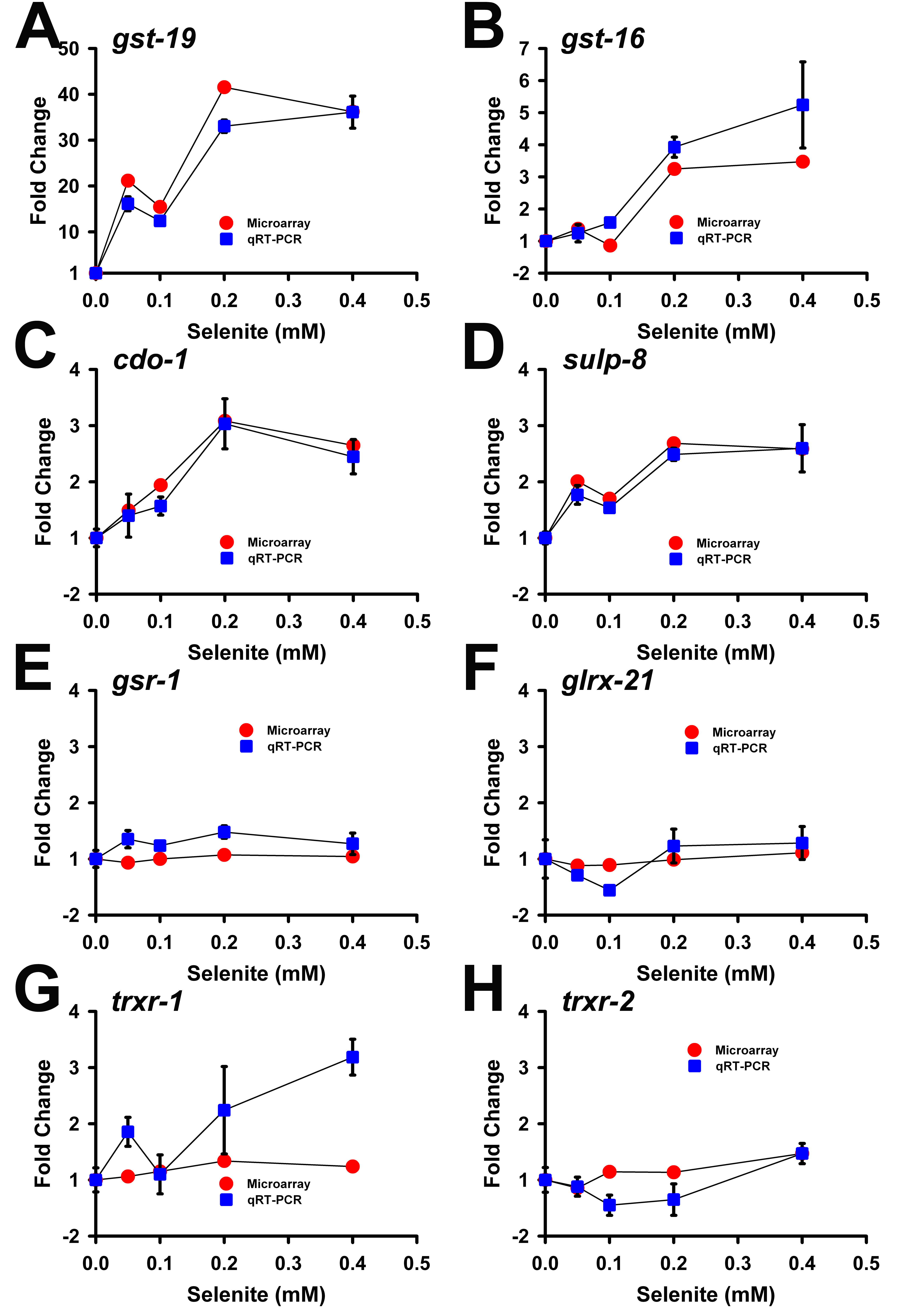

Supplement: Figure S4 — Effect of Se on relative transcript levels of highly-regulated (A–D), and marginally-regulated (E-H) oxidoreductase/sulfur metabolism genes. Transcript levels are shown as fold change (FC) relative to levels in the 0 mM Se group (FC = 1 or −1), as determined by microarray (•) and qRT-PCR (▪) on total RNA. Microarray values are FC of RMA generated expression (>100) values. qRT-PCR values are FC of mean ± SEM (n = 3) values, expressed relative to the mean of act-1 and eft-3 levels in each sample, from independent triplicate cultures grown in 0 to 0.4 mM Se. Shown are: highly-regulated genes (A) gst-19, (B) gst-16, (C) cdo-1, (D) sulp-8; marginally-regulated genes (E) gsr-1, (F) glrx-21, (G) trxr-1, (H) trxr-2 genes. Fold change for trxr-1 and trxr-2 calculated using non-normalized expression values <100. (TIF) [file pone.0101408.s004.tif]

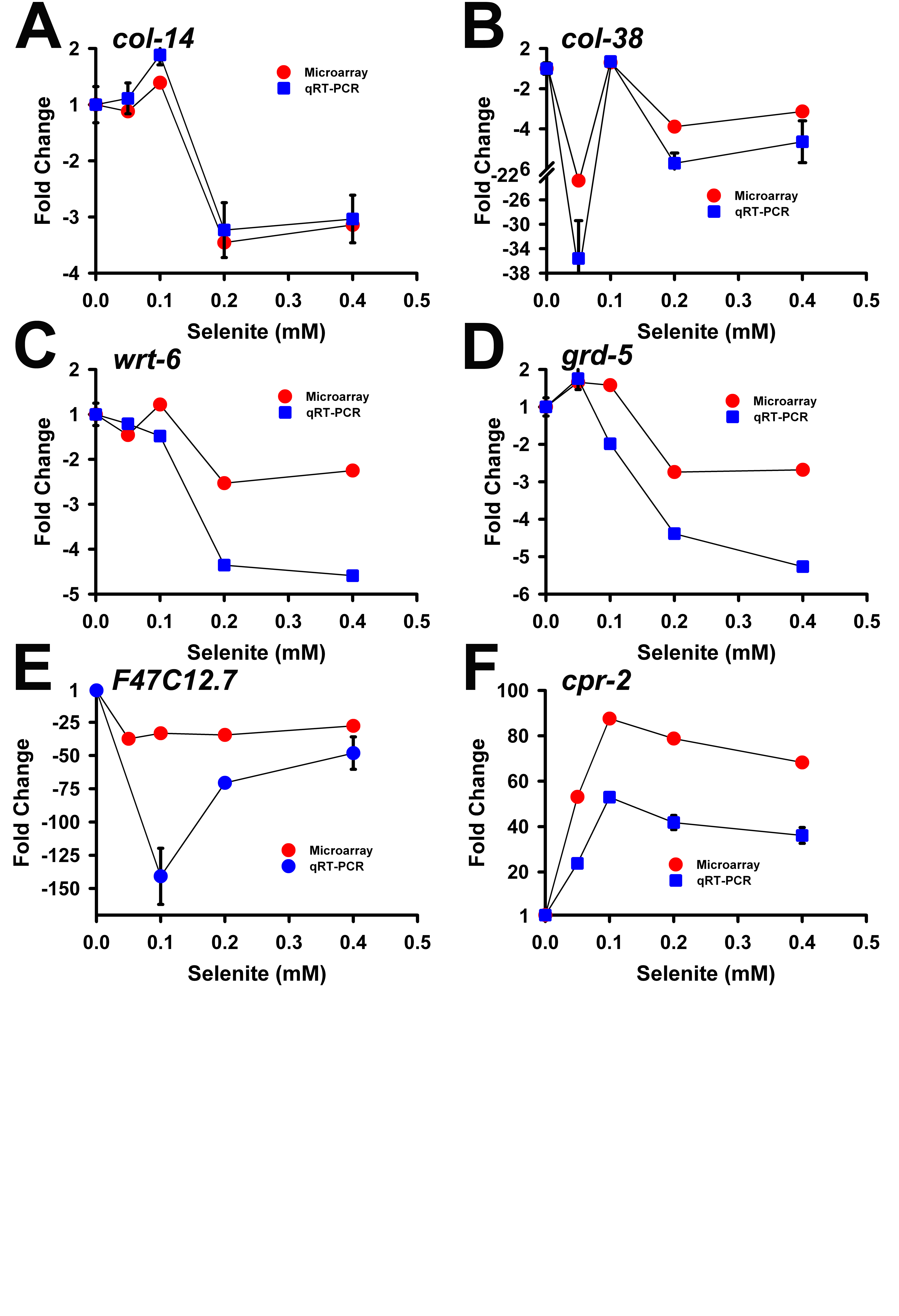

Supplement: Figure S5 — Effect of Se on relative transcript levels of collagen related (A–B), molting cycle related (C–D), and low-Se regulated (E–F) genes. Transcript levels are shown as fold change (FC) relative to levels in the 0 mM Se group (FC = 1 or −1), as determined by microarray (•) and qRT-PCR (▪) on total RNA. Microarray values are FC of RMA generated expression (>100) values. qRT-PCR values are FC of mean ± SEM (n = 3) values, expressed relative to the mean of act-1 and eft-3 levels in each sample, from independent triplicate cultures grown in 0 to 0.4 mM Se. Shown are: collagen related genes (A) col-14, (B) col-38; molting cycle related genes (C) wrt-6, (D) grd-5; low-Se regulated genes (E) F47C12.7, (F) cpr-2. (TIF) [file pone.0101408.s005.tif]
