# Supplementary material for: Toxic-Selenium and Low-Selenium Transcriptomes in Caenorhabditis elegans: Toxic Selenium Up-Regulates Oxidoreductase and Down-Regulates Cuticle-Associated Genes
Source: PLoS One. 2014 Jun 27;9(6):e101408. doi: 10.1371/journal.pone.0101408 (PMC4074201; doi:10.1371/journal.pone.0101408)
Supplement: Table S1 — Set of Toxic-Se Specific Genes. (DOC) [file pone.0101408.s006.doc]

**Supplementary Table 1. Set of Toxic-Se Specific Genes***

| **Gene Symbol** | **Probe Set ID** | **FC vs**  **0.2 mM Se^†^** | **FC vs**  **0.4 mM Se^‡^** | **Entrez Gene ID** | **WB Gene ID** |
| --- | --- | --- | --- | --- | --- |
| *scl-14* | 192465_at | 3.60 | 7.99 | 184246 | 00008625 |
| *tbb-6* | 189718_at | 3.63 | 7.10 | 179680 | 00006539 |
| *cht-1* | 171912_x_at | 2.40 | 6.44 | 180628 | 00000503 |
| *scl-5* | 192404_at | 2.48 | 5.63 | 178253 | 00008027 |
| *F07C4.6* | 180924_at | 5.03 | 5.38 | 184131 | 00017200 |
| *W06H8.2* | 182999_at | 3.11 | 4.52 | 179009 | 00021066 |
| *CELE_F20G2.1* | 188987_at | 3.73 | 3.85 | 184742 | 00008985 |
| *CELE_DC2.5* | 188800_at | 2.01 | 3.73 | 183970 | 00017082 |
| *hsp-16.1* | 189087_s_at | 3.00 | 3.54 | 179286 | hsp-16.11 |
| *mpst-3* | 182782_at | 2.96 | 3.43 | 3565829 | 00010383 |
| *F15A4.6* | 192485_at | 2.42 | 3.23 | 174887 | 00008841 |
| *clec-41* | 189915_s_at | 2.81 | 3.19 | 179797 | 00007153 |
| *T22F3.11* | 184913_s_at | 9.90 | 3.17 | 178782 |  |
| *phat-3* | 182712_at | 2.97 | 3.14 | 178809 | 00016782 |
| *Y73F4A.3* | 183055_at | 2.16 | 3.03 | 177721 | 00013516 |
| *cysl-2* | 188978_s_at | 3.15 | 2.91 | 175107 | 00010759 |
| *ech-7* | 176955_at | 5.32 | 2.89 | 173300 | 00001156 |
| *clec-42* | 193797_at | 3.80 | 2.75 | 180189 | 00008891 |
| *nhr-17* | 192635_at | 2.24 | 2.61 | 191719 | 00003616 |
| *F10E9.12* | 175175_at | 2.28 | 2.55 | 176174 | 00017361 |
| *K03H6.2* | 183309_at | 2.55 | 2.54 | 176959 | 00019368 |
| *nhr-57* | 192189_at | 2.64 | 2.50 | 178813 | 00003647 |
| *aqp-1* | 180913_at | 3.51 | 2.48 | 174190 |  |
| *clec-145* | 191784_s_at | 2.40 | 2.39 | 175003 | 00013013 |
| *CELE_C12D5.3* | 184149_at | 3.15 | 2.39 | 182534 | 00015706 |
| *CELE_F08D12.3* | 175104_s_at | 2.16 | 2.39 | 3565148 |  |
| *F35B12.3* | 182621_at | 2.74 | 2.39 | 185258 | 00009383 |
| *R06F6.7* | 186490_at | 2.46 | 2.38 | 187643 | 00011070 |
| *CELE_C55A6.6* | 190286_at | 3.18 | 2.37 | 183835 | 00008335 |
| *sre-6* | 177621_at | 3.15 | 2.33 | 185100 | 00009242 |
| *abf-5* | 177762_at | 2.39 | 2.29 | 188770 | 00000016 |
| *CELE_Y53F4B.45* | 173257_s_at | 2.34 | 2.28 | 4363034 |  |
| *C34F11.8* | 184546_s_at | 2.93 | 2.28 | 173892 | 00016417 |
| *gst-20* | 173938_s_at | 2.45 | 2.24 | 175008 | 00001768 |
| *F47B8.3* | 178790_at | 2.36 | 2.24 | 185896 | 00009804 |
| *CELE_C14C6.2* | 183566_at | 2.37 | 2.21 | 178576 | 00015756 |
| *F44E5.4* | 171941_s_at | 2.03 | 2.20 | 174805 | 00009691 |
| *cyp-33B1* | 189539_at | 2.58 | 2.18 | 182888 | 00016092 |
| *B0228.1* | 187244_at | 2.65 | 2.17 | 181861 | 00015059 |
| *kin-15* | 193979_s_at | 2.13 | 2.16 | 174498 |  |
| *ZK742.4* | 183090_s_at | 2.36 | 2.16 | 179159 | 00022814 |
| *CELE_Y75B12B.3* | 185413_at | 2.73 | 2.15 | 190717 | 00013568 |
| *R07A4.3* | 178906_at | 4.32 | 2.12 | 181270 | 00011074 |
| *ttr-21* | 190996_at | 2.35 | 2.12 | 184253 | 00008628 |
| *CELE_ZK1073.1* | 175310_s_at | 2.51 | 2.12 | 181689 | 00014213 |
| *CELE_R05D8.7* | 184068_at | 2.00 | 2.12 | 187608 | 00019885 |
| *M60.4* | 177279_at | 2.82 | 2.07 | 187465 |  |
| *unc-23* | 175047_at | 2.42 | 2.07 | 179272 |  |
| *C44B7.6* | 180212_at | 2.87 | 2.06 | 174125 |  |
| *cyp-35B1* | 189366_at | 3.19 | 2.03 | 178803 | 00019472 |
| *asah-1* | 191284_at | 2.86 | 2.02 | 173120 | 00010769 |
| *maf-1* | 173928_at | 3.17 | 2.00 | 6418592 | 00077521 |
| *F47B8.2* | 178834_at | 2.37 | 2.00 | 185895 | 00009803 |
| *E01G6.1* | 193477_s_at | -2.24 | -2.00 | 181387 | 00008449 |
| *phat-2* | 184268_s_at | -2.05 | -2.00 | 172130 | 00016733 |
| *ptr-1* | 188066_at | -2.40 | -2.02 | 191746 | 00004216 |
| *mam-2* | 183582_at | -2.56 | -2.03 | 177854 |  |
| *ZC449.2* | 191797_at | -2.12 | -2.04 | 180774 | 0002261 |
| *C26B9.2* | 181151_at | -2.29 | -2.04 | 182931 | 00016132 |
| *bus-19* | 192607_at | -2.07 | -2.04 | 179766 |  |
| *cutl-18* | 178929_at | -2.09 | -2.06 | 179565 | 00009830 |
| *zip-11* | 184700_at | -2.89 | -2.06 | 177099 | 00021082 |
| *T24C12.4* | 184236_at | -2.24 | -2.06 | 188848 | 00020766 |
| *C26B9.3* | 180439_at | -2.02 | -2.07 | 180804 | 00016133 |
| *C49F8.1* | 178577_at | -2.09 | -2.08 | 183617 | 00008213 |
| *his-48* | 172891_x_at | -2.22 | -2.09 | 178049 | 00001922 |
| *osr-1* | 191522_s_at | -2.16 | -2.09 | 172161 | 00016329 |
| *his-1* | 172733_x_at | -2.15 | -2.10 | 175027 | 00001875 |
| *CELE_C42D4.13* | 185949_at | -2.05 | -2.10 | 183405 | 00016598 |
| *col-111* | 188461_s_at | -2.13 | -2.10 | 177230 | 00000685 |
| *dpy-5* | 190360_s_at | -2.03 | -2.10 | 172197 | 00001067 |
| *CELE_F21E9.2* | 182896_at | -2.16 | -2.11 | 184781 | 00017666 |
| *nas-1* | 192577_at | -2.67 | -2.11 | 185811 | 00003520 |
| *C38C6.3* | 179600_at | -2.31 | -2.12 | 175124 | 00007996 |
| *col-131* | 188365_at | -2.27 | -2.13 | 178254 | 00000705 |
| *C30E1.9* | 174563_at | -2.04 | -2.13 | 181740 |  |
| *D2024.4* | 182111_at | -2.39 | -2.13 | 183949 |  |
| *F31D5.1* | 181614_at | -2.35 | -2.13 |  | 00017944 |
| *K03B8.6* | 178089_at | -2.52 | -2.14 | 186927 | 00010524 |
| *CELE_C02E7.7* | 172325_x_at | -2.44 | -2.14 | 178884 | 00015340 |
| *R02E4.1* | 183230_at | -2.39 | -2.15 | 187524 | 00019826 |
| *mlt-9* | 194238_x_at | -2.24 | -2.17 | 181607 |  |
| *C14F11.4* | 184320_at | -5.89 | -2.17 | 182619 |  |
| *F35D2.2* | 181659_at | -2.06 | -2.17 | 185277 | 00018040 |
| *CELE_Y11D7A.9* | 182561_at | -2.18 | -2.17 | 189434 | 00012433 |
| *CELE_F01D5.10* | 179830_at | -2.17 | -2.18 | 184059 | 00008500 |
| *R07E3.6* | 178194_at | -2.16 | -2.18 | 181240 | 00011107 |
| *C13C12.2* | 180960_at | -2.04 | -2.18 | 182568 | 00007552 |
| *F22F4.1* | 182812_at | -2.21 | -2.19 | 180868 | 00017716 |
| *CELE_K08E7.5* | 174508_s_at | -2.20 | -2.20 | 178211 |  |
| *glf-1* | 186362_s_at | -2.28 | -2.22 | 177358 | 00019154 |
| *grd-12* | 179333_at | -2.36 | -2.22 | 184079 | 00001701 |
| *nas-6* | 192447_at | -2.17 | -2.22 | 181796 |  |
| *col-139* | 189545_s_at | -5.99 | -2.23 | 178857 | 00000712 |
| *F09F9.2* | 183159_at | -2.09 | -2.25 | 184263 | 00017307 |
| *CELE_B0280.7* | 187509_at | -2.09 | -2.25 | 175992 | 00015103 |
| *spp-7* | 176857_s_at | -2.33 | -2.25 | 177326 | 00004992 |
| *mltn-12* | 178449_at | -2.74 | -2.26 | 177718 |  |
| *wrt-1* | 191180_at | -2.02 | -2.27 | 174223 | 00006947 |
| *T19A5.3* | 183911_at | -2.50 | -2.27 | 179273 |  |
| *W03D2.9* | 182019_at | -2.50 | -2.29 | 189154 | 00020983 |
| *his-11* | 172851_x_at | -2.21 | -2.29 | 175026 | 0000188 |
| *F40E10.5* | 181113_at | -2.11 | -2.29 | 185535 | 00009573 |
| *mlt-8* | 182327_at | -2.18 | -2.30 | 173448 | 00021095 |
| *H03E18.1* | 185608_at | -2.36 | -2.31 | 181103 | 00019148 |
| *C15C6.1* | 177586_at | -2.60 | -2.31 | 173098 | 00007600 |
| *CELE_F26G1.5* | 181410_s_at | -2.57 | -2.33 | 173823 | 00017841 |
| *bus-8* | 190020_at | -2.24 | -2.34 | 180821 | 00044623 |
| *lips-7* | 181956_at | -2.32 | -2.35 | 173446 |  |
| *CELE_Y45F3A.8* | 182776_at | -2.14 | -2.35 | 189910 | 00012863 |
| *F30H5.3* | 191217_s_at | -2.55 | -2.35 | 175207 | 00017937 |
| *C56C10.4* | 184220_at | -2.56 | -2.36 | 174088 | 00016962 |
| *grl-15* | 185468_at | -2.17 | -2.37 | 190707 | 00001724 |
| *col-110* | 189864_s_at | -2.68 | -2.38 | 177225 | 00000684 |
| *R02F11.1* | 185334_s_at | -2.44 | -2.38 | 178870 | 00019839 |
| *hog-1* | 188404_at | -2.12 | -2.38 | 180851 |  |
| *T06D8.10* | 188705_s_at | -2.54 | -2.38 | 174717 | 00011530 |
| *col-62* | 173767_s_at | -4.46 | -2.39 | 172494 | 00000596 |
| *nas-27* | 188410_at | -3.18 | -2.39 | 188809 | 00003545 |
| *his-12* | 172764_x_at | -2.14 | -2.39 | 172860 | 00001877 |
| *grl-7* | 173567_s_at | -2.27 | -2.39 | 179569 |  |
| *F18C5.5* | 180882_s_at | -2.08 | -2.39 | 174084 | 00017560 |
| *T06A4.3* | 189521_s_at | -2.43 | -2.40 | 171668 |  |
| *bli-5* | 177581_at | -2.12 | -2.40 | 185812 | 00000255 |
| *sqt-1* | 192674_s_at | -2.70 | -2.41 | 174731 | 00005016 |
| *acdh-6* | 187885_at | -2.46 | -2.41 | 182112 | 00015335 |
| *col-63* | 173650_s_at | -3.54 | -2.42 | 172607 | 00000639 |
| *M02G9.1* | 189891_at | -2.17 | -2.42 | 174597 |  |
| *ZK84.1* | 178652_at | -2.68 | -2.42 | 174008 | 00022649 |
| *grl-5* | 176327_s_at | -2.49 | -2.43 | 178837 | 00001714 |
| *ora-1* | 188116_at | -2.26 | -2.43 | 191739 | 00003879 |
| *mam-3* | 184682_s_at | -2.51 | -2.43 | 266652 | 00010714 |
| *mab-7* | 187901_s_at | -2.38 | -2.45 | 180440 | 00003104 |
| *suro-1* | 172989_s_at | -2.42 | -2.47 | 172557 | 00011235 |
| *C07E3.10* | 180462_at | -2.31 | -2.47 | 174599 | 00007420 |
| *H42K12.3* | 185972_at | -2.37 | -2.48 | 180474 | 00019272 |
| *C49F8.3* | 178517_at | -2.50 | -2.48 | 181459 | 00008215 |
| *W08G11.1* | 176672_at | -3.05 | -2.48 | 180099 | 00012346 |
| *phg-1* | 193258_at | -2.60 | -2.49 | 185024 | 00004017 |
| *grd-2* | 190375_at | -2.74 | -2.51 | 180349 | 00001691 |
| *C23H3.9* | 182085_at | -2.40 | -2.51 | 173391 |  |
| *nspb-2* | 172595_x_at | -3.09 | -2.51 | 185443 | 00018162 |
| *ttr-14* | 191068_at | -7.24 | -2.52 | 181272 | 00011460 |
| *his-20* | 172685_x_at | -2.14 | -2.53 | 179262 | 00001878 |
| *cut-2* | 172799_x_at | -2.86 | -2.53 | 179823 | 00009983 |
| *lon-3* | 188368_at | -2.72 | -2.53 | 179673 | 00003057 |
| *CELE_C02E7.6* | 172353_x_at | -2.61 | -2.54 | 178885 | 00015339 |
| *C14A4.9* | 178508_at | -2.48 | -2.54 | 174637 | 00007560 |
| *rol-8* | 192967_at | -2.07 | -2.55 | 174226 |  |
| *col-79* | 188675_at | -3.00 | -2.56 | 174650 | 00000655 |
| *ZK470.6* | 182490_at | -2.39 | -2.57 | 191326 | 00022747 |
| *nspb-1* | 185454_s_at | -2.75 | -2.60 | 185443 | 00018162 |
| *nas-15* | 190766_s_at | -2.06 | -2.64 | 180426 | 00003534 |
| *F53F1.4* | 172706_x_at | -2.75 | -2.64 | 179822 | 00009982 |
| *lips-3* | 191036_at | -3.35 | -2.66 | 184306 | 00008655 |
| *grd-5* | 180979_at | -2.76 | -2.67 | 179244 | 00001694 |
| *H23N18.5* | 185593_at | -2.74 | -2.67 | 186770 | 00019236 |
| *F58E6.13* | 188635_at | -3.01 | -2.68 | 179367 | 00010252 |
| *M195.2* | 177255_at | -2.38 | -2.69 | 174337 | 00010948 |
| *lips-9* | 178343_s_at | -2.80 | -2.72 | 174935 | 00010267 |
| *bli-2* | 188391_at | -3.18 | -2.73 | 191611 | 00000252 |
| *wrt-6* | 188037_at | -3.09 | -2.75 | 180638 | 00006952 |
| *M02G9.2* | 178744_at | -2.89 | -2.76 | 187405 | 00010831 |
| *M03F8.1* | 184106_at | -2.96 | -2.77 | 187429 | 00019761 |
| *pqn-32* | 180505_at | -2.78 | -2.83 | 174955 |  |
| *CELE_ZC513.1* | 183363_at | -2.86 | -2.86 | 191193 | 00022626 |
| *lrx-1* | 193409_at | -3.22 | -2.91 | 192091 | 00003075 |
| *pqn-13* | 185795_at | -2.66 | -2.96 | 178954 |  |
| *CELE_Y53F4B.27* | 184684_at | -2.92 | -2.97 | 175164 |  |
| *col-14* | 192026_at | -3.43 | -2.99 | 177559 |  |
| *Y51H7C.13* | 186760_at | -5.55 | -3.00 | 190180 | 00021791 |
| *gpdh-1* | 193659_at | -3.29 | -3.00 | 173272 | 00009824 |
| *CELE_T23F6.1* | 178038_at | -3.52 | -3.14 | 178227 | 00011949 |
| *Y51H7C.1* | 186349_at | -5.70 | -3.17 | 190173 | 00021779 |
| *col-97* | 188473_s_at | -3.83 | -3.24 | 176721 | 00000672 |
| *col-70* | 188574_s_at | -6.78 | -3.30 | 186749 | 00000646 |
| *col-70* | 188573_at | -5.08 | -3.32 | 186749 | 00000646 |
| *col-175* | 189482_s_at | -4.86 | -3.46 | 181154 | 00000748 |
| *pes-8* | 182320_at | -6.27 | -3.47 | 191742 | 00003981 |
| *M60.2* | 177368_at | -4.82 | -4.47 | 181079 | 00019779 |
| *wrt-4* | 190340_s_at | -4.06 | -4.61 | 181664 | 00006950 |
| *phat-4* | 182573_at | -2.19 | -5.11 | 178812 | 00020237 |

*Toxic-Se specific genes dataset consisting of transcripts changed by both 0.2 and 0.4 mM Se ≥2-fold relative to 0.1 mM Se, and not present in sulfur toxicity and cadmium datasets, as described in the text. Ranked by the fold-change (FC) for 0.4 mM Se dataset.

^†^Fold-change (FC) for 0.2 mM Se dataset.

^‡^Fold-change (FC) for 0.4 mM Se dataset.
