# Supplementary material for: Toxic-Selenium and Low-Selenium Transcriptomes in Caenorhabditis elegans: Toxic Selenium Up-Regulates Oxidoreductase and Down-Regulates Cuticle-Associated Genes
Source: PLoS One. 2014 Jun 27;9(6):e101408. doi: 10.1371/journal.pone.0101408 (PMC4074201; doi:10.1371/journal.pone.0101408)
Supplement: Table S2 — Gene Ontology terms enriched by toxic selenium. (DOC) [file pone.0101408.s007.doc]

**Supplementary Table 2. Gene Ontology terms enriched by toxic selenium**

| **GO ACCESSION*** | **GO Term*** | **Corrected P-value*** | **Changed Genes†** | **Total Genes‡** |
| --- | --- | --- | --- | --- |
| **GO:0031497** | chromatin assembly | 8.35E-32 | 25 | 65 |
| **GO:0040011** | locomotion | 8.35E-32 | 69 | 1418 |
| **GO:0006334** | nucleosome assembly | 8.35E-32 | 25 | 65 |
| **GO:0034728** | nucleosome organization | 8.35E-32 | 25 | 65 |
| **GO:0006333** | chromatin assembly or disassembly | 9.09E-32 | 25 | 67 |
| **GO:1990104** | DNA bending complex | 9.09E-32 | 25 | 67 |
| **GO:0000786** | nucleosome | 9.09E-32 | 25 | 67 |
| **GO:0044815** | DNA packaging complex | 1.25E-31 | 25 | 68 |
| **GO:0065004** | protein-DNA complex assembly | 8.45E-31 | 25 | 73 |
| **GO:0071824** | protein-DNA complex subunit organization | 8.45E-31 | 25 | 73 |
| **GO:0006323** | DNA packaging | 2.54E-30 | 25 | 76 |
| **GO:0000785** | chromatin | 1.64E-28 | 25 | 88 |
| **GO:0032993** | protein-DNA complex | 1.66E-26 | 25 | 104 |
| **GO:0071103** | DNA conformation change | 3.07E-25 | 25 | 116 |
| **GO:0034622** | cellular macromolecular complex assembly | 8.98E-25 | 25 | 121 |
| **GO:0006325** | chromatin organization | 4.98E-23 | 25 | 141 |
| **GO:0044427** | chromosomal part | 3.99E-22 | 25 | 153 |
| **GO:0006461** | protein complex assembly | 4.03E-21 | 25 | 168 |
| **GO:0070271** | protein complex biogenesis | 4.03E-21 | 25 | 168 |
| **GO:0044767** | single-organism developmental process | 1.04E-20 | 90 | 3882 |
| **GO:0040007** | growth | 1.07E-20 | 57 | 1452 |
| **GO:0032502** | developmental process | 1.22E-20 | 90 | 3895 |
| **GO:0002119** | nematode larval development | 1.27E-20 | 64 | 1883 |
| **GO:0002164** | larval development | 1.33E-20 | 64 | 1886 |
| **GO:0006352** | DNA-templated transcription, initiation | 1.41E-20 | 16 | 41 |
| **GO:0009791** | post-embryonic development | 2.01E-20 | 64 | 1903 |
| **GO:0065003** | macromolecular complex assembly | 2.53E-20 | 25 | 183 |
| **GO:0048856** | anatomical structure development | 2.10E-19 | 83 | 3427 |
| **GO:0051276** | chromosome organization | 4.15E-19 | 25 | 205 |
| **GO:0040008** | regulation of growth | 2.01E-18 | 58 | 1688 |
| **GO:0005694** | chromosome | 2.02E-18 | 25 | 219 |
| **GO:0045927** | positive regulation of growth | 7.92E-18 | 56 | 1614 |
| **GO:0007275** | multicellular organismal development | 4.70E-17 | 83 | 3718 |
| **GO:0071822** | protein complex subunit organization | 1.51E-16 | 25 | 262 |
| **GO:0006259** | DNA metabolic process | 3.96E-16 | 25 | 273 |
| **GO:0043933** | macromolecular complex subunit organization | 5.97E-16 | 25 | 278 |
| **GO:0044707** | single-multicellular organism process | 1.47E-15 | 85 | 4107 |
| **GO:0005811** | lipid particle | 1.77E-15 | 9 | 11 |
| **GO:0035267** | NuA4 histone acetyltransferase complex | 1.77E-15 | 9 | 11 |
| **GO:0006952** | defense response | 2.09E-15 | 17 | 98 |
| **GO:0022607** | cellular component assembly | 3.81E-15 | 25 | 302 |
| **GO:1902562** | H4 histone acetyltransferase complex | 6.36E-15 | 9 | 12 |
| **GO:0043189** | H4/H2A histone acetyltransferase complex | 6.36E-15 | 9 | 12 |
| **GO:0048518** | positive regulation of biological process | 9.19E-15 | 56 | 1891 |
| **GO:0032501** | multicellular organismal process | 1.02E-14 | 85 | 4239 |
| **GO:1902493** | acetyltransferase complex | 1.84E-14 | 9 | 13 |
| **GO:0000123** | histone acetyltransferase complex | 1.84E-14 | 9 | 13 |
| **GO:0031248** | protein acetyltransferase complex | 1.84E-14 | 9 | 13 |
| **GO:0040010** | positive regulation of growth rate | 1.46E-13 | 47 | 1429 |
| **GO:0040009** | regulation of growth rate | 1.51E-13 | 47 | 1431 |
| **GO:0044085** | cellular component biogenesis | 3.82E-13 | 25 | 371 |
| **GO:0042302** | structural constituent of cuticle | 8.27E-12 | 17 | 161 |
| **GO:0010171** | body morphogenesis | 1.18E-11 | 29 | 603 |
| **GO:0009790** | embryo development | 1.39E-10 | 62 | 2837 |
| **GO:0043565** | sequence-specific DNA binding | 1.39E-10 | 26 | 527 |
| **GO:0009792** | embryo development ending in birth or egg hatching | 1.59E-10 | 61 | 2766 |
| **GO:0005576** | extracellular region | 1.91E-10 | 22 | 368 |
| **GO:0006996** | organelle organization | 2.09E-09 | 25 | 549 |
| **GO:0044421** | extracellular region part | 8.86E-09 | 14 | 154 |
| **GO:1990234** | transferase complex | 4.63E-08 | 9 | 51 |
| **GO:0043234** | protein complex | 6.59E-08 | 26 | 701 |
| **GO:0005581** | collagen | 8.99E-08 | 8 | 38 |
| **GO:0006950** | response to stress | 1.83E-07 | 19 | 390 |
| **GO:0044420** | extracellular matrix part | 1.96E-07 | 9 | 60 |
| **GO:0005578** | proteinaceous extracellular matrix | 2.34E-07 | 10 | 83 |
| **GO:0044699** | single-organism process | 5.87E-07 | 97 | 7034 |
| **GO:0031012** | extracellular matrix | 8.56E-07 | 10 | 95 |
| **GO:0044451** | nucleoplasm part | 9.68E-07 | 9 | 72 |
| **GO:0009653** | anatomical structure morphogenesis | 1.23E-06 | 31 | 1117 |
| **GO:0044702** | single organism reproductive process | 3.51E-06 | 30 | 1105 |
| **GO:0043232** | intracellular non-membrane-bounded organelle | 3.93E-06 | 25 | 803 |
| **GO:0043228** | non-membrane-bounded organelle | 3.93E-06 | 25 | 803 |
| **GO:0040035** | hermaphrodite genitalia development | 5.41E-06 | 23 | 701 |
| **GO:0048806** | genitalia development | 6.21E-06 | 23 | 707 |
| **GO:0022414** | reproductive process | 7.41E-06 | 33 | 1349 |
| **GO:1902494** | catalytic complex | 7.70E-06 | 10 | 121 |
| **GO:0016043** | cellular component organization | 8.29E-06 | 25 | 838 |
| **GO:0005654** | nucleoplasm | 1.06E-05 | 9 | 96 |
| **GO:0003677** | DNA binding | 1.47E-05 | 26 | 927 |
| **GO:0048608** | reproductive structure development | 1.63E-05 | 24 | 810 |
| **GO:0061458** | reproductive system development | 1.63E-05 | 24 | 810 |
| **GO:0007548** | sex differentiation | 2.24E-05 | 24 | 825 |
| **GO:0032991** | macromolecular complex | 3.01E-05 | 26 | 965 |
| **GO:0071840** | cellular component organization or biogenesis | 3.07E-05 | 25 | 904 |
| **GO:0016539** | intein-mediated protein splicing | 3.07E-05 | 4 | 9 |
| **GO:0030908** | protein splicing | 3.07E-05 | 4 | 9 |
| **GO:0042329** | structural constituent of collagen and cuticulin-based cuticle | 3.07E-05 | 4 | 9 |
| **GO:0048513** | organ development | 4.04E-05 | 25 | 919 |
| **GO:0042303** | molting cycle | 4.80E-05 | 13 | 269 |
| **GO:0018996** | molting cycle, collagen and cuticulin-based cuticle | 4.80E-05 | 13 | 269 |
| **GO:0048731** | system development | 9.63E-05 | 26 | 1032 |
| **GO:0044446** | intracellular organelle part | 1.08E-04 | 25 | 973 |
| **GO:0060102** | collagen and cuticulin-based cuticle extracellular matrix | 1.10E-04 | 4 | 12 |
| **GO:0065007** | biological regulation | 1.14E-04 | 63 | 4089 |
| **GO:0003006** | developmental process involved in reproduction | 1.34E-04 | 24 | 921 |
| **GO:0050789** | regulation of biological process | 1.45E-04 | 62 | 4024 |
| **GO:0005198** | structural molecule activity | 1.47E-04 | 17 | 504 |
| **GO:0000003** | reproduction | 2.15E-04 | 41 | 2214 |
| **GO:0044422** | organelle part | 2.69E-04 | 25 | 1029 |
| **GO:0090304** | nucleic acid metabolic process | 5.46E-04 | 25 | 1073 |
| **GO:0006351** | transcription, DNA-templated | 0.001 | 16 | 532 |
| **GO:0051604** | protein maturation | 0.001 | 4 | 22 |
| **GO:0016485** | protein processing | 0.001 | 4 | 22 |
| **GO:0032774** | RNA biosynthetic process | 0.001 | 16 | 542 |
| **GO:0031981** | nuclear lumen | 0.004 | 9 | 207 |
| **GO:0008150** | biological_process | 0.005 | 106 | 9347 |
| **GO:0040002** | collagen and cuticulin-based cuticle development | 0.009 | 6 | 98 |
| **GO:0008233** | peptidase activity | 0.009 | 12 | 392 |
| **GO:0042335** | cuticle development | 0.009 | 6 | 99 |
| **GO:0016491@** | oxidoreductase activity | 0.012 | 10 | 607 |
| **GO:0055114@** | oxidation-reduction process | 0.012 | 10 | 652 |
| **GO:0060103** | collagen and cuticulin-based cuticle extracellular matrix part | 0.014 | 2 | 4 |
| **GO:0060106** | cortical layer of collagen and cuticulin-based cuticle extracellular matrix | 0.014 | 2 | 4 |
| **GO:0007365** | periodic partitioning | 0.014 | 2 | 4 |
| **GO:0007367** | segment polarity determination | 0.014 | 2 | 4 |
| **GO:0034654** | nucleobase-containing compound biosynthetic process | 0.018 | 16 | 685 |
| **GO:0010467** | gene expression | 0.020 | 20 | 979 |
| **GO:0070013** | intracellular organelle lumen | 0.020 | 9 | 261 |
| **GO:0043233** | organelle lumen | 0.021 | 9 | 263 |
| **GO:0016540** | protein autoprocessing | 0.022 | 2 | 5 |
| **GO:0031974** | membrane-enclosed lumen | 0.028 | 9 | 274 |
| **GO:0019438** | aromatic compound biosynthetic process | 0.039 | 16 | 742 |
| **GO:0040014** | regulation of multicellular organism growth | 0.040 | 11 | 409 |
| **GO:0044428** | nuclear part | 0.041 | 9 | 291 |
| **GO:0003676** | nucleic acid binding | 0.044 | 26 | 1525 |
| **GO:0018130** | heterocycle biosynthetic process | 0.044 | 16 | 754 |
| **GO:0044271** | cellular nitrogen compound biosynthetic process | 0.045 | 16 | 756 |

*Gene Ontology (GO) accession numbers and terms significantly enriched, with associated *P*-values, determined when down-regulated genes and up-regulated genes in the toxic-Se specific data set were separately subjected to GeneSpring gene ontology analysis (*P* <0.05), resulting in 125 terms for down-regulated genes and 2 terms for up-regulated genes.

†Number of genes involved in this process in the set of toxic-Se specific genes

‡Number of genes involved in this process in the GeneChip C. elegans Genome Array

@Terms for up-regulated genes.
