# Supplementary material for: Toxic-Selenium and Low-Selenium Transcriptomes in Caenorhabditis elegans: Toxic Selenium Up-Regulates Oxidoreductase and Down-Regulates Cuticle-Associated Genes
Source: PLoS One. 2014 Jun 27;9(6):e101408. doi: 10.1371/journal.pone.0101408 (PMC4074201; doi:10.1371/journal.pone.0101408)
Supplement: Table S3 — Set of Low-Se Specific Genes. (DOT) [file pone.0101408.s008.dot]

**Supplementary Table 3. Set of Low-Se Specific Genes***

| **Gene Symbol** | **Probe Set ID** | **FC vs**  **0.05 mM Se^†^** | **FC vs**  **0.1 mM Se^‡^** | **Entrez Gene ID** | **WB Gene ID** |
| --- | --- | --- | --- | --- | --- |
| *F47C12.7* | 186033_at | 37.44 | 33.23 | 185919 | 00018550 |
| *ZK6.11* | 177783_at | 4.48 | 5.01 | 191209 |  |
| *cav-1* | 173403_s_at | 3.50 | 2.76 | 177815 |  |
| *F49D11.6* | 185390_at | 2.70 | 2.70 | 186035 | 00018630 |
| *F54E2.1* | 180150_at | 2.58 | 2.65 | 178742 | 00018823 |
| *inx-2* | 173200_s_at | 2.49 | 2.49 | 181318 | 00002124 |
| *spp-4* | 181156_at | 3.59 | 2.28 | 188266 | 00004989 |
| *F21D5.3* | 191407_s_at | 2.15 | 2.26 | 177680 |  |
| *car-1* | 194165_s_at | 3.22 | 2.25 | 173158 | 00012484 |
| *ttr-50* | 190831_s_at | 3.51 | 2.13 | 178092 | 00010234 |
| *K12H4.7* | 175962_s_at | 3.62 | 2.12 | 176136 |  |
| *CELE_C46C2.5* | 187646_s_at | 5.46 | 2.11 | 177742 | 00008113 |
| *F08F3.6* | 183194_s_at | 4.63 | 2.01 | 178925 | 00017263 |
| *wrt-10* | 184676_s_at | -2.32 | -2.01 | 174224 | 0000695 |
| *lips-10* | 177829_at | -2.40 | -2.01 | 174345 | 00008803 |
| *T13C2.2* | 179776_at | -2.43 | -2.03 | 188470 | 00020478 |
| *Y43E12A.2* | 184368_at | -2.01 | -2.05 | 177993 | 00012794 |
| *F13D11.4* | 193849_at | -2.50 | -2.06 | 184416 | 00017429 |
| *CELE_F54B8.1* | 179246_s_at | -2.05 | -2.07 | 186199 | 00010016 |
| *ins-5* | 188319_at | -3.76 | -2.07 | 191686 | 00002088 |
| *ZK596.1* | 177660_at | -2.04 | -2.10 | 177982 | 00014006 |
| *C44E12.1* | 185117_at | -2.80 | -2.10 | 181023 | 00016657 |
| *lact-3* | 188063_s_at | -2.11 | -2.11 | 174644 | 00010897 |
| *H19M22.3* | 175035_at | -2.10 | -2.12 | 175383 |  |
| *CELE_F49F1.5* | 180727_at | -2.02 | -2.13 | 186062 | 00018645 |
| *lpr-1* | 186995_at | -2.12 | -2.17 | 171648 | 00002393 |
| *fkb-7* | 187987_at | -2.37 | -2.22 | 172963 | 00001432 |
| *F52H3.5* | 179402_at | -2.12 | -2.24 | 174562 | 00009947 |
| *glt-5* | 192556_s_at | -2.99 | -2.25 | 174530 | 00001623 |
| *CELE_Y43F8B.2* | 172578_x_at | -2.06 | -2.32 | 180278 |  |
| *nnt-1* | 193024_at | -2.24 | -2.34 | 180884 | 00003778 |
| *F45E1.4* | 182286_at | -2.02 | -2.35 | 185793 | 00018465 |
| *CELE_T02B11.4* | 186124_at | -2.15 | -2.37 | 187981 | 00020156 |
| *T05C1.3* | 181888_at | -3.35 | -2.43 | 188114 | 00020250 |
| *CELE_Y39G8B.7* | 180410_at | -3.39 | -2.44 | 189765 | 00012727 |
| *dhs-4* | 175398_s_at | -2.37 | -2.45 | 172810 | 00000968 |
| *CELE_M162.5* | 177250_at | -3.02 | -2.46 | 180300 | 00010931 |
| *F11E6.4* | 179570_at | -2.67 | -2.49 | 184358 | 00008708 |
| *H40L08.2* | 174393_at | -2.92 | -2.58 | 13224091 |  |
| *sqrd-1* | 178043_at | -2.68 | -2.63 | 178374 |  |
| *ugt-25* | 184602_at | -2.77 | -2.64 | 172087 | 00015692 |
| *PDB1.1* | 177951_s_at | -2.23 | -2.70 | 181016 |  |
| *F41C3.1* | 181414_at | -3.48 | -2.76 | 185595 | 00018267 |
| *T05E7.1* | 182338_at | -2.04 | -2.77 | 172309 | 00020258 |
| *ins-4* | 188314_at | -3.56 | -2.78 | 191685 | 00002087 |
| *Y45F10D.6* | 184224_at | -2.35 | -2.84 | 189925 | 00012886 |
| *ubc-23* | 191748_at | -2.19 | -2.89 | 182989 | 00006718 |
| *K01A2.3* | 183231_at | -3.42 | -3.00 | 173420 | 00019278 |
| *CELE_F59A7.2* | 183014_at | -5.48 | -3.03 | 178682 | 00019090 |
| *W04A8.4* | 178654_s_at | -2.98 | -3.21 | 173253 | 00012239 |
| *CELE_F25D1.3* | 178006_at | -3.49 | -3.35 | 179468 | 00009108 |
| *Y42G9A.3* | 184843_at | -3.20 | -3.58 | 175865 |  |
| *mtl-2* | 188487_s_at | -4.52 | -3.88 | 179899 | 00003474 |
| *cah-4* | 189628_at | -2.51 | -3.89 | 181478 |  |
| *clec-8* | 186194_s_at | -4.52 | -5.07 | 174893 | 00012582 |
| *dpy-17* | 181096_at | -5.37 | -5.34 | 175696 | 00001076 |
| *R08E5.1* | 182504_at | -4.90 | -5.74 | 178794 | 00019961 |
| *R08E5.3* | 182747_at | -8.45 | -7.19 | 178795 | 00019963 |
| *cpr-2* | 189469_at | -53.01 | -87.46 | 185355 | 00000782 |

*Low-Se specific genes dataset consisting of transcripts changed by both 0 mM Se ≥2-fold relative to 0.05 and 0.1 mM Se, as described in the text. Ranked by the fold-change (FC) relative to 0.1 mM Se dataset.

^†^Fold-change (FC) relative to 0.05 mM Se dataset.

^‡^Fold-change (FC) relative to 0.1 mM Se dataset.
